# Allogeneic Vγ9Vδ2 T-cell immunotherapy exhibits promising clinical safety and prolongs the survival of patients with late-stage lung or liver cancer

**DOI:** 10.1038/s41423-020-0515-7

**Published:** 2020-09-16

**Authors:** Yan Xu, Zheng Xiang, Mohammed Alnaggar, Léonce Kouakanou, Jiawei Li, Junyi He, Jiashuang Yang, Yi Hu, Yan Chen, Li Lin, Jianlei Hao, Jingxia Li, Jibing Chen, Man Li, Qingling Wu, Christian Peters, Qinghua Zhou, Jianshuang Li, Yingqing Liang, Xiaohua Wang, Baohui Han, Meili Ma, Dieter Kabelitz, Kecheng Xu, Wenwei Tu, Yangzhe Wu, Zhinan Yin

**Affiliations:** 1 The First Affiliated Hospital, Faculty of Medical Science, Jinan University, Guangzhou, 510632 Guangdong PR China; 2 Zhuhai Precision Medical Center, Zhuhai People’s Hospital (Zhuhai Hospital Affiliated with Jinan University), Jinan University, Zhuhai, 519000 Guangdong PR China; 3 The Biomedical Translational Research Institute, Jinan University, Guangzhou, 510632 Guangdong PR China; 4 Department of Paediatrics and Adolescent Medicine, Li Ka Shing Faculty of Medicine, University of Hong Kong, Hong Kong, PR China; 5 Institute of Immunology, Christian-Albrechts-University Kiel, Kiel, Germany; 6 Fuda Cancer Hospital, Faculty of Medical Science, Jinan University, Guangzhou, 510665 Guangdong PR China; 7 Shanghai Chest Hospital, Shanghai Jiao Tong University, Shanghai, PR China; 8 Present Address: Tongji Chibi Hospital, Tongji Medical College, Huazhong University of Science and Technology, Chibi, Hubei PR China

**Keywords:** Allogeneic γδ T cells, New expansion formula, Cell therapy, Liver cancer, Lung cancer, Humanized mice, Translational immunology, Cancer immunotherapy, Immunotherapy

## Abstract

Vγ9Vδ2 T cells are promising candidates for cellular tumor immunotherapy. Due to their HLA-independent mode of action, allogeneic Vγ9Vδ2 T cells can be considered for clinical application. To apply allogeneic Vγ9Vδ2 T cells in adoptive immunotherapy, the methodology used to obtain adequate cell numbers with optimal effector function in vitro needs to be optimized, and clinical safety and efficacy also need to be proven. Therefore, we developed a novel formula to improve the expansion of peripheral γδ T cells from healthy donors. Then, we used a humanized mouse model to validate the therapeutic efficacy of expanded γδ T cells in vivo; furthermore, the expanded γδ T cells were adoptively transferred into late-stage liver and lung cancer patients. We found that the expanded cells possessed significantly improved immune effector functions, including proliferation, differentiation, and cancer cell killing, both in vitro and in the humanized mouse model. Furthermore, a phase I clinical trial in 132 late-stage cancer patients with a total of 414 cell infusions unequivocally validated the clinical safety of allogeneic Vγ9Vδ2 T cells. Among these 132 patients, 8 liver cancer patients and 10 lung cancer patients who received ≥5 cell infusions showed greatly prolonged survival, which preliminarily verified the efficacy of allogeneic Vγ9Vδ2 T-cell therapy. Our clinical studies underscore the safety and efficacy of allogeneic Vγ9Vδ2 T-cell immunotherapy, which will inspire further clinical investigations and eventually benefit cancer patients.

## Introduction

Cancer is one of the leading lethal diseases worldwide because of its high incidence and mortality. In 2018, 18.1 million new cancer cases and 9.6 million cancer deaths were estimated to occur worldwide.^1^ Therefore, developing new antitumor strategies for reducing mortality rates and improving patients’ quality of life are urgently needed. In this respect, the introduction of checkpoint inhibitors to unleash the activity of tumor-reactive T cells has been a milestone in cancer immunotherapy.^2^ Among various newly emerging treatment strategies, adoptive immune cell transfer therapy (ACT) has attracted great attention. ACT potentially includes αβ T-cell (e.g., CD8^+^ T cells and CAR-T cells),^3–5^ natural killer (NK) cell-^6–8^ and gamma delta (γδ) T-cell-based^9–15^ immunotherapy.

γδ T cells, specifically the Vγ9Vδ2 subset, which is the dominate subset among γδ T cells in human peripheral blood, recognize target cells in a human leukocyte antigen (HLA)-independent manner. Moreover, γδ T cells can directly kill target cells without the involvement of dendritic cells (DCs) and perform dual functional roles in antitumor and anti-infective immunity. Vγ9Vδ2 T cells recognize pyrophosphates secreted by many microbes or overproduced by malignant cells in the context of butyrophilin 3A1 molecules.^16,17^ Importantly, endogenous production of pyrophosphates can be stimulated by nitrogen-containing aminobisphosphonates, such as zoledronate (ZOL), leading to potent activation of γδ T cells.^18^ Such advantages enable γδ T cells to rapidly respond against malignant transformation and pathogenic stress.^19^ For instance, γδ T cells are the earliest producers of IFN-γ in the tumor microenvironment^20^ and during spinal cord injury.^21^ γδ T cells utilize a variety of surface receptors and cytokines, such as NKG2D, TRAIL, FASL, TNF-α, IFN-γ, Granzyme B, and perforin, to initiate cytotoxicity against cancer cells.^11–13,22,23^ In addition, given that different subtypes of γδ T cells possess diverse functional signatures, investigations on total γδ T cells in the context of the tumor microenvironment or immunotherapy tend to produce controversial results; for example, there are inconsistent views^24^ on the intratumoral γδ T-cell signature as the most favorable prognostic biomarker^25^ of cancers and discussions on the pro- and antitumor activities of γδ T cells.^22,26^ Nevertheless, Vγ9Vδ2 T cells have promising clinical value and advantages for tumor immunotherapy. Furthermore, a few clinical studies based on γδ T cells as a therapy for both solid tumors and hematological malignancies have been performed, as summarized in Wilhelm’s review.^27^ However, all previous clinical trials based on autologous γδ T cells derived from cancer patients^27^ reported only limited clinical efficacy.^26^ Therefore, we previously reported that the adoptive transfer of allogeneic Vγ9Vδ2 T cells can improve the immune functions of cancer patients, including CD4 T-cell, CD8 T-cell, and NK-cell numbers; these improvements were accompanied by the complete relief of recurrent liver cancer in a patient.^9^ For the first time, this work showed that allogeneic Vγ9Vδ2 T cells could be used as a new therapeutic strategy for at least certain types of cancer.

An allogeneic Vγ9Vδ2 T-cell-based transfer approach might thus be a new strategy for antitumor immune cell therapy, making it possible to provide off-the-shelf immune cells to cancer patients without concerns about adverse effects resulting from graft-versus-host disease (GVHD) or cytokine storm due to the unique immunological properties of Vγ9Vδ2 T cells. At present, however, there are at least two scientific barriers that need to be overcome to effectively apply allogeneic Vγ9Vδ2 T cells in clinical tumor therapy. One is the need to optimize the existing cell expansion methodology to obtain a large quantity of Vγ9Vδ2 T cells with relatively strong antitumor activity. A hurdle here is the limited number of Vγ9Vδ2 T cells in the peripheral blood, which usually account for 2–4% of peripheral blood T cells. Cell expansion strategies have been continuously investigated, and expansion methodologies were summarized in the review by the Wang group.^28^ The common protocol is the use of interleukin (IL)-2 plus a phosphate antigen, such as ZOL,^29–31^ bromohydrin pyrophosphate,^32^ isopentenyl pyrophosphate,^18^ or (E)-4-hydroxy-3-methyl-but-2-enyl pyrophosphate.^17,33^ In addition, the feeder cell-based expansion method for Vγ9Vδ2 T cells has also been investigated.^34^ Moreover, gene modification-related technologies have been used to generate potent γδ T cells, including T-cell receptor (TCR) transfer or chimeric antigen receptor (CAR) expression.^14,35,36^ Previously, our investigation on γδ T-cell expansion discovered that vitamin C could greatly promote the proliferation and effector functions of Vγ9Vδ2 T cells.^29^ Vitamin C is a potent antioxidant and epigenetic modifier that has been shown to modulate T-cell differentiation.^37^ However, the development of a new optimal expansion formula for Vγ9Vδ2 T cells resulting in stronger cytotoxicity remains a pressing need.

On the other hand, autologous γδ T cells have very limited efficacy in cancer therapy,^26^ and one of the crucial causes is the systemic dysfunction of immune cells in cancer patients. Allogeneic strategies (healthy donor-derived γδ T cells) have been theoretically considered for years since these cells possess better antitumor activity and achieve a more promising clinical prognosis in cancer therapy than autologous cells. Therefore, the second scientific barrier is that the clinical safety of allogeneic Vγ9Vδ2 T-cell transfer needs to be scientifically proven. This can only be achieved through clinical studies in cancer patients.

In the present work, to resolve these two scientific barriers, we first developed a new formula (NF) for Vγ9Vδ2 T-cell expansion in vitro, which consisted of ZOL, IL-2, IL-15, and vitamin C. We found that, compared with the old formula (OF cells; ZOL plus IL-2), our NF could better promote γδ T-cell proliferation and differentiation. Compared with OF cells, γδ T cells expanded with the NF (termed NF cells) had significantly higher expression of costimulatory molecules, a substantially stronger cellular energy metabolism capability, and considerably higher levels of effector molecules (IFN-γ, TNF-α, and NKG2D) and the degranulation molecule CD107a, resulting in enhanced cytotoxicity against various cancer cell lines in vitro. NF cells significantly inhibited lung tumor growth in humanized mice and greatly prolonged mouse survival. Second, through a phase I clinical investigation, we demonstrated the clinical safety of 414 allogeneic NF-cell infusions in 132 late-stage cancer patients and observed sound clinical efficacy in 18 late-stage lung and liver cancer patients. Overall, our present work moved the allogeneic strategy a step forward and will inspire an increasing number of clinical studies on allogeneic Vγ9Vδ2 T cells in cancer immunotherapy.

## Materials and methods

### Expansion of Vγ9Vδ2 T cells ex vivo for in vitro and in vivo experiments

Human peripheral blood mononuclear cells (PBMCs) were isolated from healthy donors using a Ficoll-Paque-based density gradient centrifugation protocol. The cells were cultured in RPMI 1640 medium supplemented with 10% FBS and antibiotics. To activate cells, ZOL (50 μM working concentration, Sigma) was added to the culture medium on day 0. Recombinant human IL-2 (100 IU/mL) (Beijing Four Rings Bio-Pharm Co.), recombinant human IL-15 (100 IU/mL), and vitamin C (70 μM) (Sigma) were included in the medium as well (NF). After 12–14 days of culture, cells were used in experiments. For comparison, the OF (ZOL + IL-2) was also used to culture cells to perform control experiments (in vitro and in mice). In this paper, the NF-expanded γδ T cells were termed NF cells, and the OF-expanded γδ T cells were termed OF cells. For in vitro comparison of biological function between NF cells and OF cells, cells were derived from the same donors.

### Vγ9Vδ2 T-cell functional analysis

Vγ9Vδ2 T cells were stimulated with 5 μg/mL plate-bound anti-CD3 antibodies and 1 μg/mL soluble anti-CD28 antibodies (free mAbs) for 6 h in the presence of 5 μg/mL Brefeldin A. Then, the cells were stained with anti-human CD3-APC-H7 (BD Biosciences, clone: SK7), anti-human TCR Vδ2-PerCP (BioLegend, clone: B6), and anti-human CD107a-APC (BD Biosciences, clone: H4A3) antibodies. After staining for surface markers, the cells were fixed and permeabilized using Lysing Solution (BD Biosciences) and Permeabilizing Solution (BD Biosciences), respectively. Subsequently, the cells were stained with anti-human IFN-γ-PE-Cy7 (BD Biosciences, clone: B27), anti-human TNF-α-PE (BD Biosciences, clone: MAb11), and their corresponding isotype controls (APC-conjugated mouse IgG1, κ isotype control (clone: MOPC-21); PE-Cy™7-conjugated mouse IgG1, κ isotype control RUO (clone: MOPC-21); and PE-conjugated mouse IgG1, κ isotype control (clone: MOPC-21), all from BD Biosciences). Then, the cells were analyzed with a BD FACS Verse, and the data were analyzed with FlowJo. In addition, cell cycle, cell proliferation (Ki-67, BioLegend, clone: Ki-67), cell differentiation (CD45RA, BioLegend, clone: HI100), CD27 (BioLegend, clone: O323), mitochondrial, and costimulatory molecule (CD80, BioLegend, clone: 2D10; CD86, BioLegend, clone: BU63) analyses were performed following standard protocols.

### In vitro cytotoxicity assay

To determine the cytotoxicity of Vγ9Vδ2 T cells (effector, E), several human cancer cell lines (target, T) were used to perform killing assays, including a lung cancer cell line (A549), an acute T-cell leukemia cell line (Jurkat T), a breast cancer cell line (MCF-7), a human Burkitt lymphoma cell line (BJAB), a leukemia cell line (K562), and the human Burkitt’s lymphoma cell lines Raji and Daudi. Cancer cells were prestained with 2 μM CFSE. Effector cells and target cells were cocultured at different E:T ratios (1:1, 1:5, and 1:10) at 37 °C for 6 h. The cell apoptosis of cancer cells was analyzed using flow cytometry and PI (0.2 µg/mL) staining. As a control, CD8^+^ T cells and PBMCs from unrelated donors were used to test the allogeneic reaction of Vγ9Vδ2 T cells, and normal human umbilical vein endothelial cells were used to test the cytotoxicity of Vγ9Vδ2 T cells against normal cells.

### Mitochondrial detection

Fourteen-day-expanded OF cells and NF cells were stained with MitoTracker™ Red CMXRos (20 nM) (Invitrogen), DAPI (Abcam), and an anti-Vδ2-FITC antibody (BioLegend). The number and distribution of mitochondria were observed by confocal microscopy (Leica). The fluorescence intensity of mitochondria was analyzed by flow cytometry. Fourteen-day-expanded OF cells and NF cells were stained with MitoTracker Green (20 nM) (Beyotime), an anti-CD3-APC-H7 antibody (BioLegend), and an anti-Vδ2-PE antibody (BioLegend).

### Metabolism assays

Oxygen consumption rates (OCRs) and extracellular acidification rates (ECARs) were measured using the Seahorse XF-96 Extracellular Flux Analyzer (Agilent) following the standard protocol. The reagents (Agilent) included XF Base medium containing 10 mM glucose, 2 mM l-glutamine, 1 mM sodium pyruvate, 1 μM oligomycin, 0.25 μM FCCP, 0.5 μM rotenone, and antimycin A.

### Western blotting

For western blot experiments, the procedure was as follows. In brief, cells were washed with cold phosphate buffered saline (PBS) three times. Then, 100 μL/10^7^ cells ice-cold RIPA buffer supplemented with a protease inhibitor cocktail (Roche) and phosphatase inhibitor cocktails A and B (Selleck) was added. The cell suspensions were incubated for 30 min on ice, and then the lysates were clarified by centrifugation for 10 min at 12,000 RPM and 4 °C. The supernatants were transferred to fresh tubes and stored on ice or frozen at −20 or −80 °C. The concentration of protein was measured using a spectrophotometer. Twenty micrograms of protein was used for western blotting. SDS-PAGE was used for total protein separation, and the proteins were electrotransferred to PVDF membranes (Roche). The membranes were blocked with 5% BSA (Sigma Aldrich) for 2 h at room temperature. All western blotting antibodies, such as antibodies against Bcl2, Caspase 3, FasL and α-Tubulin, were from Cell Signaling Technology. The working dilution of the anti-α-tubulin antibody used was 1:5000, and that of the other antibodies was 1:1000. The membranes were incubated with the indicated primary antibody overnight at 4 °C, followed by washing three more times with TBST for 10 min. This was followed by incubation with an appropriate HRP-linked secondary antibody (horse anti-mouse or goat anti-rabbit IgG) for 2 h at room temperature. The membranes were then washed three more times with TBST. The resulting films generated from the membranes were subjected to semiquantitative analysis with a Bio-Rad ChemiDoc XRS system (Bio-Rad Corporation, USA).

### Library construction for RNA-seq and sequencing procedures

A total of 5 × 10^6^ OF cells or NF cells were collected from three samples per group on day 14 for RNA extraction. Total RNA was isolated using an RNeasy mini kit (Qiagen, Germany). Strand-specific libraries were prepared using a TruSeq® Stranded Total RNA Sample Preparation kit (Illumina, USA) following the manufacturer’s instructions. Briefly, ribosomal RNA was removed from the total RNA using Ribo-Zero rRNA removal beads. Following purification, the mRNA was fragmented into small pieces using divalent cations at 94 °C for 8 min. The cleaved RNA fragments were copied into first-strand cDNA using a reverse transcriptase and random primers. This was followed by second-strand cDNA synthesis using DNA Polymerase I and RNase H. These cDNA fragments then underwent an end repair process, the addition of a single “A” base, and ligation of adapters. The products were then purified and enriched with PCR to create the final cDNA library. Purified libraries were quantified with the Qubit® 2.0 Fluorometer (Life Technologies, USA) and validated with an Agilent 2100 bioanalyzer (Agilent Technologies, USA) to confirm the insert size and calculate the mole concentration. A cluster was generated by cBot with the library diluted to 10 pM, and then sequenced on the Illumina HiSeq 2500 (Illumina, USA). Library construction and sequencing were performed at Shanghai Biotechnology Corporation.

### Data analysis for gene expression

Sequencing raw reads were preprocessed by filtering out rRNA reads, sequencing adapters, short-fragment reads, and other low-quality reads. We used TopHat v2.0.9 to map the clean reads to the human HG19 reference genome with two mismatches. After genome mapping, Cufflinks v2.1.1 was run with reference annotation to generate FPKM values for known gene models. Differentially expressed genes were identified using Cuffdiff. The *p* value significance threshold in multiple tests was set by the false discovery rate (FDR). Fold changes were also estimated according to the FPKM of each sample. Differentially expressed genes were selected using the following filter criteria: FDR ≤ 0.05 and fold change ≥ 2.

### In vivo evaluation of the antitumor activity of Vγ9Vδ2 T cells using humanized mice

The establishment of humanized mouse model and related experimental procedures referred to our previously published work in *Cancer Cell*^38^ and *Journal of Experimental Medicine*.^39^ Six- to eight-week-old Rag2^−/−^γc^−/−^ mice were maintained in the Laboratory Animal Unit at the University of Hong Kong. All animal studies were approved by the Committee on the Use of Live Animals in the Teaching and Research, University of Hong Kong and performed in compliance with the guidelines for the use of experimental animals set by this committee. According to the published protocol for establishing a humanized mouse model,^38,39^ huPBMCs were isolated from buffy coat preparations of whole blood from healthy donors. Rag2^−/−^γc^−/−^ mice were treated with 200 μL liposomes (Liposoma B.V., Netherlands) by intravenous injection (i.v.) one day before transplantation. Sublethally irradiated mice were then transplanted i.p. with 30 × 10^6^ huPBMCs. Four weeks after huPBMC transplantation, the mice with successful reconstitution of human immune system and were subsequently used to establish GFP-A549 lung cancer mice model. GFP-A549 cells (1 × 10^5^/mouse) were injected subcutaneously into humanized mice. Subsequently, these lung cancer-bearing mice were randomly divided into three groups (*n* = 5) on day 7. Then, the three groups of mice received PBS (100 μL), OF-expanded allogeneic Vγ9Vδ2 T cells or NF-expanded allogeneic Vγ9Vδ2 T cells (5 × 10^6^ cells/mouse; cells with >90% purity were resuspended in 100 μL PBS) at days 7, 10, 13, 16, and 19 by i.v. For such mouse experiments, infused Vγ9Vδ2 T cells were prepared from the same donor by using frozen huPBMCs. Tumor volume was measured every 7 days from the first day of cell injection, and the animal survival rate was recorded every day. Tumor size was calculated as (length × width^2^)/2. Mice with subcutaneous tumors more than 17 mm in diameter were killed and counted as dying.

### In vivo tracking of DiR-labeled Vγ9Vδ2 T cells

Vγ9Vδ2 T cells were stained with DiR (1,1′-dioctadecyl-3,3,3′,3′-tetramethylindotricarbocyanine iodide, Invitrogen), and then these DiR-labeled cells were adoptively transferred intravenously into subcutaneous GFP-A549 tumor-bearing mice. The migration and accumulation of Vγ9Vδ2 T cells were depicted and analyzed with a TM2 in vivo imaging system (CRI Maestro) at the indicated time points.

### Ethics

Our relevant clinical studies have been registered on the United States clinical trial website (https://www.clinicaltrials.gov/; ID: NCT03183206, NCT03183219, NCT03183232, and NCT03180437). All clinical studies were approved by the ethics committee of the Fuda Cancer Hospital affiliated with Jinan University (Guangzhou), and each enrolled patient signed an informed consent form.

### Recruitment of patients

The rules for recruitment of patients included the following: (1) patients were diagnosed with middle-advanced lung or liver cancer by histology and cytology. (2) The patient life expectancy was at least 12 weeks with long-term follow-up possible. (3) Imaging examination showed the largest tumor length was ideally ≤2 cm; otherwise, patients needed to receive tumor ablation first. (4) Surgery and chemotherapy were deemed unsuitable in any of the following situations: Karnofsky performance status score ≥ 70, white blood cell count ≥ 3 × 10^9^/L, neutrophil count ≥ 2 × 10^9^/L, hemoglobin level ≥ 90 g/L, platelet count ≥ 100 × 10^9^/L, prothrombin time (international normalized ratio) ≥ 1.5 (with no severe coronary heart disease, myelosuppression, respiratory disease, and/or acute/chronic infection), level 3 hypertension, and adequate hepatic function (total bilirubin < 75 μmol/L, direct bilirubin < 39 μmol/L, and Child-Pugh score of A or B) and renal function (serum creatinine < 130 μM, serum urea < 10 mm). (5) Patients could be male or female within the age range of 18–60 years. (6) Patients were willing to receive γδ T-cell therapy.

Exclusion criteria included (1) systemic diseases, such as severe coagulopathy, or other viral infections, bacterial infections, or infectious diseases; (2) hepatic and renal insufficiency, atrial fibrillation, and cardiac load tests that found inducible myocardial ischemia or uncontrollable angina; (3) other diseases, such as endocrine system diseases and mental illness, and a lack of suitability for lymphocyte treatment after diagnosis and evaluation by physicians; and (4) patient request to quit.

### Clinical observation index

In this work, clinical examination and follow-up included (1) clinical indicators (appetite, sleep, pain, etc.); (2) immune function evaluation; (3) biochemical indicators, routine blood tests, coagulation function, and tumor markers; and (4) enhanced CT, MRI, or PET/CT examination.

### Vγ9Vδ2 T-cell expansion for clinical immunotherapy and clinical application

To perform large-scale generation of cells for the purpose of clinical cancer immunotherapy, Vγ9Vδ2 T cells were expanded ex vivo using the procedures described for the in vitro and in vivo experiments above. It should be mentioned here, however, that all procedures, including cell isolation, expansion, and quality control (cell purity, expression of cytotoxicity and inhibitory marker molecules, etc.) were performed in a certified cell culture facility (good manufacturing practices, GMP). During cell expansion, every 3 days during cell culture, 100 µL cell culture medium was taken and tested for mycoplasma, chlamydia, bacteria, fungi, and endotoxin. Cell expansion generally took 12 days to reach the scale of 10^8^. Cells that could not pass quality control were removed for harmless disposal.

Afterwards, 1–2 × 10^8^ NF cells cultured from PBMCs collected from healthy donors (each batch of cells was freshly generated from random donors) were adoptively transferred intravenously into patients. The cell purity used for clinical treatment was ≥90%. The rate of cell expansion was ~500. It should be noted here that 10% of non-Vγ9Vδ2 T cells mainly consisted of B, NK, NKT, CD4, CD8, and Vδ1-T cells according to our analyses.^9^ On the day of transfusion, cells were collected and washed five times with physiological saline to remove cytokines and serum. Then, the cells were resuspended in physiological saline to prepare a cell suspension, and the cell density was adjusted to 1 × 10^8^/50 mL. The cell dosage used in our clinical study was based on the previously published literature.^9^ Patients received cell therapy once every 2–3 weeks for the first five infusions, then once per month or every 2 months.

Follow-up and clinical observations were performed as scheduled in the protocols. The patients received plain CT and enhanced CT 2 weeks before treatment and were reexamined every 3 or 6 months during follow-up, and the last follow-up was completed in June 2020. It should be mentioned that late-stage patients who had tumor sizes > 2 cm received irreversible electroporation (IRE), I^125^, cryoablation, or combination therapy (refer to Supplementary Tables) prior to cell infusion; otherwise, only cell therapy was applied. Furthermore, even though an ablation therapy such as IRE, I^125^, and/or cryoablation was applied, the survival time of enrolled late-stage cancer patients was clinically estimated to be only ~3–8 months.

### Data analyses and statistics

Statistical analyses were performed using GraphPad Prism (GraphPad Software, Inc.). Experimental repetitions are noted in the corresponding figure legend. All results are expressed as the mean ± SEM (standard error of the mean). Significant differences in cell proliferation, the cell cycle, and metabolism among groups, as well as tumor incidence and tumor size among PBS-, NF-cell-, and OF-cell-treated groups, were analyzed by ANOVA. Apoptosis, Ki-67, cytokine, and killing assays were analyzed by a paired *t*-test. The *p* value of the difference in survival was determined with the Kaplan–Meier log-rank test. Statistical significance is described in detail in the figure legends.

## Results

### The new formula promoted the proliferation and differentiation of Vγ9Vδ2 T cells

**Fig. 1:**
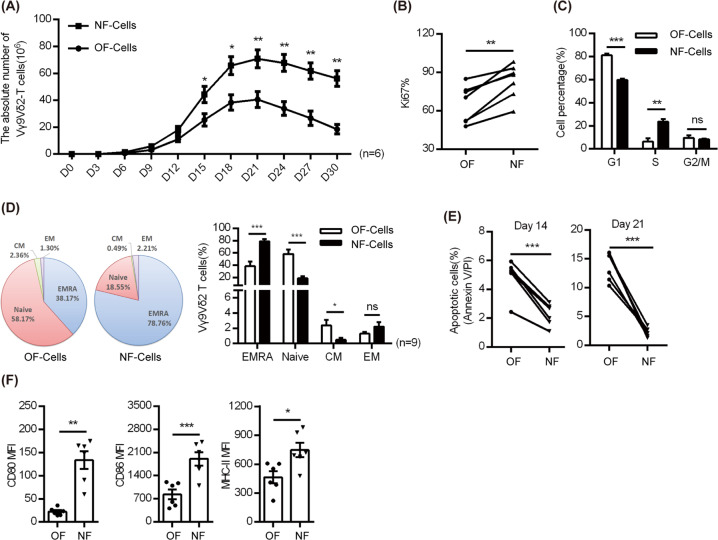
New formula (NF) significantly promoted the proliferation and differentiation and reduced the apoptosis of Vγ9Vδ2 T cells. **a** Absolute cell counts on various indicated days were calculated by using flow cytometry (anti-TCR Vδ2-PE and anti-CD3-FITC antibodies). Here, 5 × 10^6^ PBMCs were used in each group to test two culture medium formulas (six individual donors). The time points analyzed were days 0, 3, 6, 9, 12, 15, 18, 21, 24, 27, and 30. **b** Cell proliferation was evaluated by staining for Ki-67 (seven individual donors). **c** The new formula significantly promoted cell mitosis, as evidenced by G1-phase reduction and S-phase augmentation (six individual donors). **d** The new formula (NF) could induce the differentiation of Vγ9Vδ2 T cells, as shown by significant increases in the percentages of effector memory (EM; CD45RA^−^CD27^−^) and terminally differentiated (EMRA; CD45RA^+^CD27^−^) Vγ9Vδ2 T cells and significant decreases in the percentages of naive (CD45RA^+^CD27^+^) and central memory (CD45RA^−^CD27^+^) cells, compared with the old formula (OF) (six individual donors). **e** Cell apoptosis was significantly decreased with the new formula compared to the old formula. Cell apoptosis was assayed on day 14 (normal culture time) and day 21 (long-term culture) (six individual donors). **f** The new formula enhanced the expression levels of costimulatory molecules on γδ T cells (six individual donors). MFI mean fluorescence intensity. The levels of costimulatory molecules (CD80, CD86, and HLA class-II) on Vγ9Vδ2 T cells were measured by flow cytometry, showing that NF cells expressed significantly higher levels of these costimulatory molecules than OF cells. Error bars represent the standard error of the mean (SEM). *p < 0.05; **p < 0.01; ***p < 0.001

To evaluate the expansion ability of Vγ9Vδ2 T cells using the NF (ZOL + IL-2 + IL-15 + vitamin C), we performed absolute counting of Vγ9Vδ2 T cells and assessments of cell proliferation and differentiation markers. We found that compared with medium containing the OF (ZOL + IL-2), NF culture medium could greatly promote cell proliferation, evidenced by significantly higher absolute cell numbers (Fig. 1a) and Ki-67 expression (Fig. 1b). RNA sequencing also showed that the NF significantly upregulated the expression of proliferation-related genes compared with the OF (Supplementary Fig. 1A), such as CDC25B, CDKN1B, PAN2, LUC7L3, and LYAR. Furthermore, we found that NF medium could significantly promote cell mitosis by reducing the G1-phase cell proportion while augmenting the S-phase cell proportion (Fig. 1c). To analyze cellular differentiation, cells were analyzed for CD45RA and CD27 expression, and the results showed a significant increase in the terminally differentiated effector memory (EMRA; CD45RA^+^CD27^−^) Vγ9Vδ2 T-cell proportion, while at the same time, significant decreases in the naive (CD45RA^+^CD27^+^) and central memory (CM; CD45RA^−^CD27^+^) cell proportions were observed. No significant variation was observed for effector memory (EM; CD45RA^−^CD27^−^) cells (Fig. 1d). These results revealed that the NF could significantly enhance the proliferation and differentiation of Vγ9Vδ2 T cells.

### The new formula reduced cell apoptosis, and NF cells expressed higher levels of costimulatory molecules than OF cells

Since the NF elevated the percentage of EMRA cells, it was important to investigate whether the NF could delay cell apoptosis. This is of significance because the antiapoptotic ability determines how long expanded cells can persist in vivo. We found that the NF could notably reduce the apoptosis of expanded Vγ9Vδ2 T cells (Fig. 1e). Thus, on day 14 of culture, the apoptosis rate of NF cells was ~1.9- to 3-fold lower than that of OF cells, and on day 21 of culture, the apoptosis rate of NF cells was ~3.8- to 12.3-fold lower than that of OF cells. RNA-seq results further revealed that compared with the OF, the NF could significantly downregulate the expression of apoptosis-related genes, such as CASP8, CD44, CDC25B, Smad7, and H1F0 (Supplementary Fig. 1B). Together, these results suggested that the NF could maintain long-term cell viability by reducing apoptosis. This was also supported by increased BCL2 and reduced caspase 3 and FASL protein levels in γδ T cells expanded in NF medium (Supplementary Fig. 1C). Furthermore, we also analyzed the expression levels of costimulatory molecules on Vγ9Vδ2 T cells using flow cytometry. We found that NF cells expressed significantly higher levels of costimulatory molecules, including CD80, CD86, and MHC-II, than OF cells, as shown in Fig. 1f.

### NF cells exhibited stronger cellular energy metabolism than OF cells

**Fig. 2:**
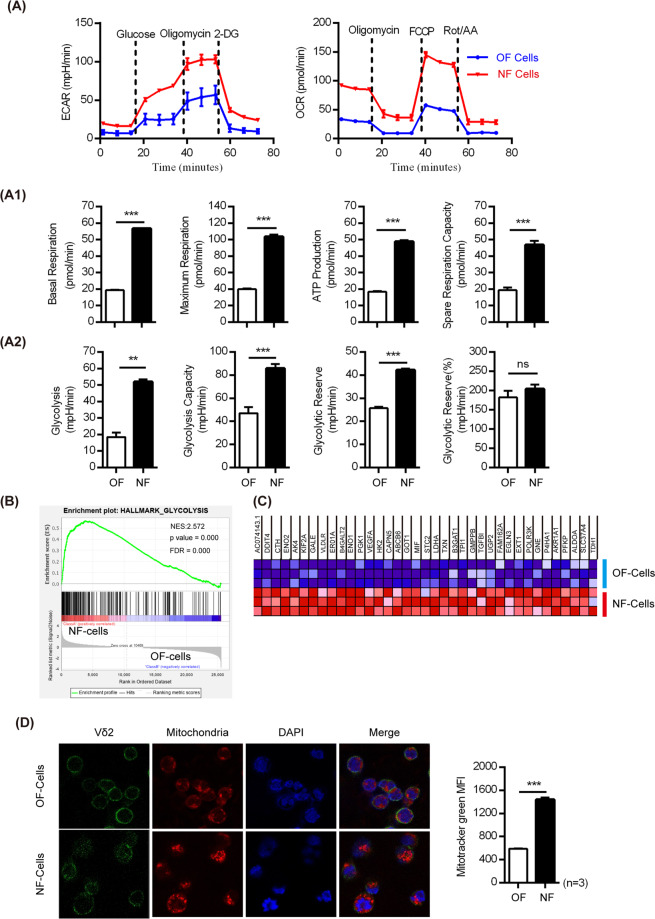
NF cells possess stronger cellular energy metabolism, including both mitochondrial respiration and glycolysis, than OF cells. **a** Metabolic results were obtained with a Seahorse XF analyzer. The ECAR was assessed after the addition of 25 mM glucose (gluc) and in response to the metabolic inhibitors oligomycin (oligo) and 2DG. O_2_ consumption rates (OCRs) were measured in real time under basal conditions and in response to the indicated mitochondrial inhibitors (three repetitions, mean ± SEM). A1 Quantitative comparisons of mitochondrial functions, including basal respiration, maximum respiration, ATP production and spare respiration capacity, between NF cells and OF cells were performed. A2 Quantitative comparisons of glycolytic metabolism, including glycolysis, the glycolytic capacity and the glycolytic reserve, between NF cells and OF cells were performed. Data are representative of three independent experiments. **b** Gene set enrichment analysis (GSEA) identified similarities between OF cells and NF cells. The expression of glycolysis-related genes was upregulated in NF cells. **c** The heat map illustrates the expression of glycolysis-related genes in NF cells versus OF cells. Red: upregulated; blue: downregulated. **d** The distribution of mitochondria was observed by confocal microscopy, showing that NF cells possessed higher mitochondrial fluorescence than OF cells. Cells were stained by using MitoTracker (red), DAPI (blue) and an anti-Vδ2 antibody (green) for confocal visualization. **e** The fluorescence intensity of mitochondria was statistically analyzed by flow cytometry (three repetitions, mean ± SEM). *p < 0.05; **p < 0.01; ***p < 0.001

Since the NF could promote cell differentiation by enhancing the proportion of T_EMRA_ cells (Fig. 1d) and the reported literature^40^ shows that glycolysis predominates in activated and effector CD8^+^ T cells, it was of interest to reveal whether the NF alters the cellular metabolism of Vγ9Vδ2 T cells. Therefore, we analyzed differences in cellular energy metabolism, including mitochondrial respiration and glycolysis, between NF cells and OF cells. Mitochondrial respiration and glycolysis were recorded through measurement of the OCR and ECAR, respectively, by a Seahorse XF analyzer. As shown in Fig. 2a, we discovered that both the OCR and the ECAR were dramatically elevated in NF cells. For instance, OCR parameters, including basal respiration, maximum respiration, ATP production, and spare respiration capacity, were all significantly enhanced (Fig. 2A1). For the ECAR, glycolysis, the glycolytic capacity, and the glycolytic reserve were notably augmented as well (Fig. 2A2). Moreover, gene expression profile analysis via gene set enrichment analysis revealed significant upregulation of glycolysis-related gene expression in NF cells as well (Fig. 2b, c).

Because NF cells and OF cells had a significant difference in mitochondrial respiration, we visualized mitochondrial alterations using confocal microscopy (Fig. 2d), which showed a higher fluorescence signal for mitochondria in NF cells than in OF cells. Moreover, the fluorescence intensity of mitochondria was analyzed by flow cytometry (right graph in Fig. 2d), and the results indicated that NF cells had a remarkably higher fluorescence signal than OF cells. Together, these results demonstrated that NF cells had notably higher mitochondrial contents than OF cells.

### NF cells expressed a higher level of effector molecules and had stronger in vitro killing ability against cancer cells than OF cells

**Fig. 3:**
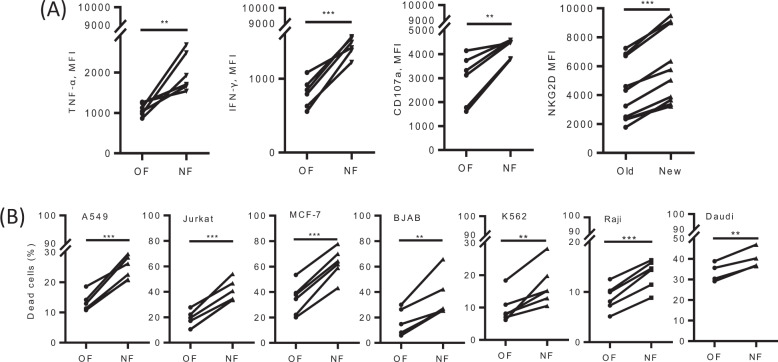
New formula upregulates the expression levels of CD107a, NKG2D, IFN-γ, and TNF-α and potentiates the in vitro cytotoxicity of Vγ9Vδ2 T cells. **a** TNF-α, IFN-γ, CD107a, and NKG2D levels were measured after 14 days following stimulation for 6 h with anti-CD3 (5 μg/mL) and anti-CD28 (1 μg/mL) antibodies. Data are shown as the mean fluorescence intensity (MFI). **b** The new formula could significantly enhance the in vitro antitumor cytotoxicity of Vγ9Vδ2 T cells, as shown by killing assay results obtained for A549, Jurkat, MCF-7, BJAB, K562, Raji, and Daudi target cells. The Vγ9Vδ2 T cells were cocultured for 6 h with CFSE-labeled tumor cells at a 10:1 ratio. Each linked line between OF and NF in all graphs represents an individual γδ T-cell line. **p < 0.01; ***p < 0.001

Next, we analyzed the expression of effector molecules, including cytokines and surface receptors. The NF considerably upregulated the expression of molecules related to activation (NKG2D), effector functions (IFN-γ and TNF-α), and degranulation (CD107a) (Fig. 3a). Then, the in vitro killing capacity against normal cells and different types of cancer cells (the lung cancer cell line A549, acute T-cell leukemia cell line Jurkat, breast cancer cell line MCF-7, human Burkitt lymphoma cell line BJAB, chronic myeloid leukemia cell line K562, and human Burkitt’s lymphoma cell lines Raji and Daudi) was investigated. As shown in Supplementary Fig. 2A, NF cells had no cytotoxicity towards normal cells, such as CD8 T cells and PBMCs. However, NF cells exerted significantly stronger in vitro antitumor cytotoxicity against cancer cell lines, at both low (1:1) (Supplementary Fig. 2B) and higher (10:1) effector:target ratios (Fig. 3b), than the OF, exhibiting an ~1- to 3-fold increase.

### In vivo validation of the antitumor activity of NF cells in humanized mice

**Fig. 4:**
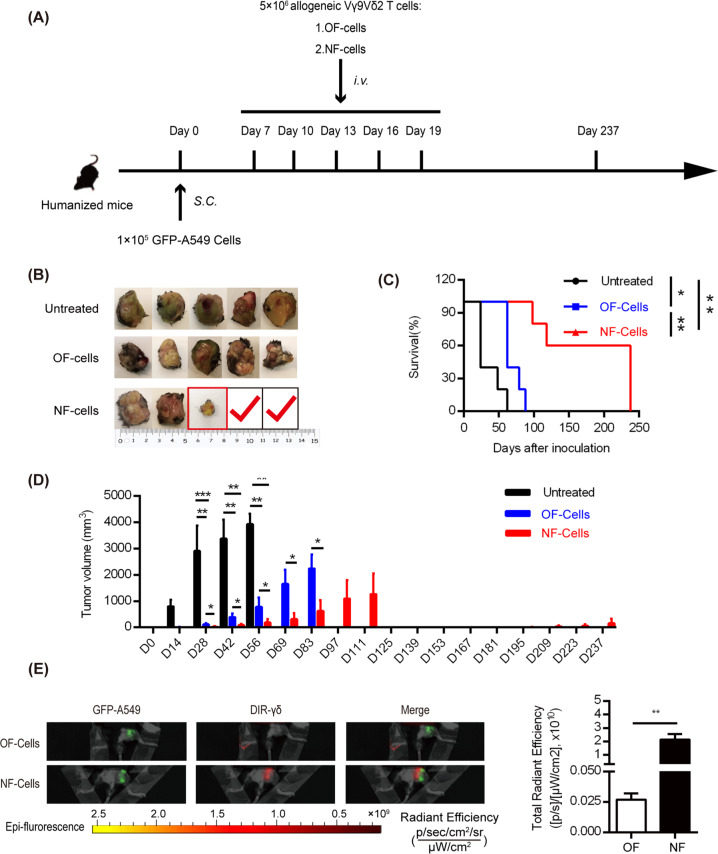
NF-γδ T cells displayed potent antitumor activity in humanized mice. **a** The protocol for establishing lung cancer in humanized mice and evaluating the therapeutic effects of NF cells and OF cells. Seven days after inoculation of GFP-549 cells into humanized mice, NF cells and OF cells were adoptively transferred i.v. into the humanized mice. Mice treated with PBS were used as the control. **b** Macroscopic tumor inspection of tumors from untreated, OF-cell-treated, and NF-cell-treated mice. For the NF-cell-treated group, one mouse had a recurrent tumor on day 125, and two mice were tumor-free when sacrificed on day 238. **c** Survival curves of the three groups, suggesting that NF cells could significantly prolong survival time of tumor-bearing mice. **d** Tumor volume comparisons among the untreated, OF-cell-treated, and NF-cell-treated groups, showing that NF cells have optimal therapeutic effects on tumor development. **e** Live imaging to visualize the colocalization of A549 cancer cells and γδ T cells in humanized mice (views of the lower limb region of mice). NF cells or OF cells were prelabeled with DiR (red color) and then transferred i.v. into mice. The live imaging data indicated that only NF cells (red) visually colocalized with tumor cells (green) when monitored at 48 h post infusion. Data are presented as the mean ± SEM. *p < 0.05; **p < 0.01; ***p < 0.001

Since the in vitro results demonstrated that NF cells were functionally more robust than OF cells, it was of great importance to validate the antitumor activity of NF cells in vivo. Here, we used humanized mice inoculated with the human lung cancer cell line GFP-A549. On day 7 post inoculation, NF cells or OF cells were intravenously transferred into the tumor-bearing mice according to the protocol (Fig. 4a), and PBS was used as a control. Upon macroscopic inspection (Fig. 4b), we found that the OF cells mildly slowed tumor growth and prolonged mouse survival (blue curve in Fig. 4c). In contrast, the NF cells eradicated tumors in two mice that were sacrificed on day 238 (one mouse had a recurrent tumor on day 125, highlighted by the red square). With respect to survival, we found that NF-cell therapy could strikingly prolong the mouse survival time (red curve in Fig. 4c). Notably, the untreated and OF-cell-treated mice survived less than 62 days and 88 days, respectively. In contrast, for the NF-cell-treated mice, one mouse survived for 98 days, another survived for 118 days, and the remaining three mice were still alive when they were sacrificed on day 238 (two were tumor-free, and one had a recurrent tumor develop on day 125) (Fig. 4b, c).

Moreover, we also tracked tumor growth by measuring tumor volume (Fig. 4d) and discovered that γδ T-cell treatment could slow tumor growth in all mice. Furthermore, NF cells could significantly inhibit tumor progression compared with OF cells. In addition, we performed live imaging to visualize the colocalization of A549 cancer cells and γδ T cells in humanized mice, and the results showed that only NF cells (red color) were visually colocalized with tumor cells (green color) at 48 h post infusion (Fig. 4e), implicating with stronger homing ability of NF cells. In addition, significantly more NF cells than OF cells accumulated at the tumor site, as indicated by the higher fluorescence signal of DIR-NF cells (the right graph in Fig. 4e). In addition, it should be noted that NF-cell treatment had no side effects on organs, including the lungs, liver, kidneys, and heart, showing the safety of γδ T cells (Supplementary Fig. 3).

### Immunotherapy of late-stage liver and lung cancer patients using allogeneic Vγ9Vδ2 T cells expanded with the new formula

For late-stage malignant liver and lung cancer patients, there is no current treatment to either prolong their survival or improve their quality of life. Given that Vγ9Vδ2 T cells recognize and kill cancer cells in an HLA-independent manner, NF-expanded allogeneic Vγ9Vδ2 T cells from healthy donors were used to clinically treat 132 late-stage cancer patients, including those with lung, liver, pancreatic, breast (NCT03183232, NCT03183219, NCT03183206, and NCT03180437) or other types of tumors, with a total number of 414 allogeneic NF-cell infusions (Supplementary Table 1). The procedures for cell expansion to administration are shown in Supplementary Fig. 4. According to clinical observations, allogeneic Vγ9Vδ2 T cells produced no significant adverse effects (e.g., immune rejection, cytokine storm, or GVHD effects) (Supplementary Fig. 5 and Supplementary Table 1).

Among the enrolled patient volunteers, those patients who had a tumor size > 2 cm received IRE, I^125^, cryoablation, or combination therapy prior to cell infusion; otherwise, only cell therapy was applied. Among those 132 cancer patients, most received only 1–4 cell infusions. Since the primary purpose of our clinical study was to validate the safety of allogeneic Vγ9Vδ2 T cells, the inclusion criteria were not strictly followed when volunteer patients wanted to receive cell therapy.

**Fig. 5:**
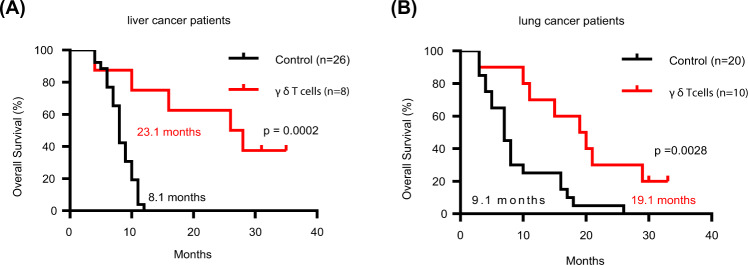
Overall survival of patients with advanced liver cancer or lung cancer infused with allogeneic Vγ9Vδ2 T cells expanded using the new formula. **a**, **b** Overall survival curves were plotted by comparing γδ T-cell-treated patients and patients not treated with γδ T cells. The survival time of the γδ T-cell-treated patients was calculated starting from the date of the first infusion until June 2020. For the liver cancer group, the median survival time of the untreated patients was 8.1 months; in contrast, the median survival time of the γδ T-cell-treated patients was 23.1 months. *P* = 0.0002. For the lung cancer group, the median survival time of the untreated patients was 9.1 months; however, the median survival time was 19.1 months for the γδ T-cell-treated patients. *P* = 0.0028

According to clinical records, only 8 liver cancer and 10 lung cancer patients received ≥5 NF-cell infusions; these 18 patients were then followed, and their clinical information and survival data are shown in Table 1 and Fig. 5. Seven of the eight liver cancer patients (Fig. 5a) and nine of the ten lung cancer patients (Fig. 5b) survived ≥10 months. Representative CT images also illustrated the therapeutic efficacy of NF cells (Supplementary Fig. 6). In our latest follow-up in June 2020, the results showed that three liver cancer and two lung cancer patients were still alive between 30 and 35 months after adoptive γδ T-cell therapy (gray columns in Fig. 5b).

**Table 1:** Summary of the clinical outcomes of patients with metastatic liver or lung cancer treated with allogeneic γδ T cells

	Patient number	Sex	Age (year)	(Diagnosis/phase)	Infusion time	Treatment	Times of γδ T cells treatment	Clinical response	PA date	Survival (month)
Liver cancer patients										
1	507882	M	47	Low differentiated hepatocellular carcinoma (T4N0M1 IV stage)	2017.6–2018.3	γδ + IRE	7	PA	2019.10	28
2	507524	F	56	Hepatic tubular cell carcinoma (cT3N1M0 IVA stage)	2017.8–2017.10	γδ + I^125^	6	SD	Live	35
3	507590	M	66	Hepatocellular carcinoma (T3bN1M0 IV stage)	2017.7–2017.10	γδ	5	PA	2018.5	10
4	004701	M	66	Hepatocellular carcinoma of hepatobiliary duct (T2N0M0 II stage)	2017.8–2018.4	γδ + IRE	7	PA	2019.10	26
5	507770	M	31	Hepatocellular carcinoma of hepatobiliary duct (TxN1M1 IV stage)	2017.8–2018.4	γ^δ^ + I^125^	13	CR	Live	35
6	508940	M	48	Hepatocellular carcinoma of hepatobiliary duct (T4N0M1 IV stage)	2017.9–2017.12	γδ + IRE + CYRO	6	PA	2018.1	4
7	375046	M	29	Hepatocellular carcinoma (TxNXM0 IV stage)	2017.11–2018.4	γδ	5	PD	Live	31
8	509410	F	40	Hepatocellular carcinoma (pT4NxMx III stage)	2018.2–2018.5	γδ + IRE	6	PA	2019.6	16
Lung cancer patients										
1	507937	M	68	Left lung adenocarcinoma (cT3NxM1 IV stage)	2017.5–2017.10	γδ + IRE + CYRO	6	PA	2018.12	19
2	507936	F	61	Left lung adenocarcinoma (pT3N2M1 IV stage)	2017.5–2018.4	γδ + CYRO	12	PA	2019.10	29
3	508259	F	65	Right lung cancer (cTxNxM1 IV stage)	2017.6–2017.12	γδ	6	PA	2018.9	15
4	508328	M	67	Right lung cancer (TXN2M1 IV stage)	2017.7–2017.12	γδ + I^125^	6	PA	2018.6	11
5	508144	F	55	Left lung small cell lung cancer (IV stage)	2017.4–2017.9	γδ	8	PA	2018.2	10
6	507497y	F	58	Left lung squamous cell carcinoma (pT2N2M1 IV stage)	2017.9–2018.6	γδ + I^125^	8	PD	Live	33
7	509166	F	64	Left upper lung adenocarcinoma post operation (T4N0M0 III A stage)	2017.12–2018.2	γδ	6	PA	2019.9	21
8	509331	F	57	Right lung adenocarcinoma (T4N0M0)	2017.12–2018.5	γδ	6	SD	Live	30
9	509459	F	47	Lung cancer (TXN2M1 IV stage)	2018.2–2018.4	γδ	7	PA	2018.5	3
10	508354	M	52	Right lung squamous carcinoma (T4N1M1 IV stage)	2017.4–1017.12	γδ + I^125^	6	PA	2018.12	20

Patients with advanced liver or lung cancer were intravenously infused with NF cells (1–2 × 10^8^ cells per treatment, purity >90%). Among the liver cancer patients, eight patients received ≥5 NF-cell infusions. These eight patients were followed until June 2020, and three of them were alive with current survival times of 30–35 months. The follow-up evaluation was performed according standards of the Response Evaluation Criteria in Solid Tumors (RECIST). Among the lung cancer patients, ten patients received ≥5 NF-cell infusions. These ten patients were followed until June 2020 as well. Nine of these ten patients survived ≥10 months, and two of them were still alive.

*M* male, *F* female, *PA* passed away, *PD* progressive disease, *CR* complete response, *SD* stable disease

Moreover, immune-phenotype analysis of patients was performed before and after γδ T-cell treatment. Changes in the proportions of immune cells (CD4^+^, CD8^+^, NK, and γδ cells) and functional subsets in lung cancer and liver cancer patients are shown in Supplementary Fig. 7. These results showed that γδ T-cell therapy could greatly improve immune cell parameters by regulating the frequencies of these immune cells. Specifically, the administration of γδ T cells was associated with increases in the proportions of CD28^+^ CD4/CD8 T cells (CD3^+^CD4^+^/CD8^+^CD28^+^), naive CD8^+^ T cells (CD3^+^CD8^+^CCR7^+^CD45RA^+^), central memory CD8 T cells (CD3^+^CD8^+^CCR7^+^CD45RA^−^), and NKp30^+^ γδ T cells (CD3^+^γδT^+^NKp30^+^). These increases were accompanied by decreases in the proportions of aged CD4/CD8 T cells (CD3^+^CD4/CD8^+^CD28^−^), senescent CD8^+^ T cells (CD3^+^CD8^+^CD28^−^CD57^+^), and PD-1^+^ γδ T cells (CD3^+^γδ^+^PD-1^+^). Surprisingly, we also observed a decrease in the peripheral NK-cell (CD3^−^CD56^+^) proportion. This might be due to peripheral NK-cell migration to the tumor site induced by the infused γδ T cells, but the mechanism remains to be scientifically identified. It should be emphasized here that we did not examine such phenotypes through on-bench experiments, which thus could only provide indications on changes in immune function in the context of allogeneic Vγ9Vδ2 T-cell therapy. In addition, we also followed the persistence of adoptively transferred γδ T cells in the cancer patients, making use of the differential expression of HLA-A2 on donor and recipient cells. HLA-A2-positive (i.e., donor-derived) lymphocytes were still detectable (7.23%) 10 days after cell infusion (Supplementary Fig. 8).

## Discussion

In addition to surgery, chemotherapy, and radiotherapy, immunotherapy is now considered the fourth therapeutic strategy for malignant tumors. Among immunotherapy strategies, cellular immunotherapy has received global attention from researchers. Currently, there are various strategies with genetically modified (e.g., CAR-αβ T) cells,^3–5^ nonmodified (e.g., γδ T, NK, DC-CIK) cells^6,7,9,14,41^ as well as CAR-NK^42–44^ and CAR-γδ T^14,35,36,45^ cell-related technologies that can be used in cancer immunotherapy. Even though gene modification approaches, such as CAR technology, have achieved ground-breaking success in CD19+ diseases,^3–5^ the application of such a strategy in solid tumors is still under continuous investigation. Notably, the published literature has intensively reviewed the clinical potential of Vγ9Vδ2 T cells as a new strategy for cancer immunotherapy;^13–15,22,23^ for instance, previous clinical trials on non-small cell lung cancer,^10,46^ hepatocellular carcinoma^47^ renal cell carcinoma,^48–51^ and myeloma^52^ proved the feasibility of Vγ9Vδ2 T cells in tumor immunotherapy. However, limited clinical efficacy^26^ makes Vγ9Vδ2 T cells of modest value in clinical investigation and application.

To our knowledge, the main reason leading to the limited clinical efficacy of Vγ9Vδ2 T cells includes at least two aspects. The first issue is that it remains a challenge to figure out how to potentiate the antitumor cytotoxicity of nonmodified immune cells. The second issue has been seen in all past clinical trial studies using the autologous γδ T-cell strategy. Since tumorigenesis commonly results in long-term immune depression, cancer cells have evolved strategies for immune escape and tolerance induction through various pathways, including upregulation of PD-L1 expression and secretion of inhibitory molecules, such as TGF-β and IL-10.^53^ This immunosuppression can result in intrinsic defects in immune cells because of the high level of lactic acid along with the low levels of glucose and oxygen present in the tumor microenvironment,^54^ rendering cytotoxic immune cells unable to either recognize cancer cells or initiate cytotoxicity against cancer cells. Functional defects might be the main reason why autologous immune cells have achieved only modest clinical efficacy. Therefore, developing new allogeneic immune cell-based therapeutic strategies could overcome the shortcomings of autologous cell therapy.

γδ T cells are promising candidates for ACT due to their unique immunological advantages for tumor immunotherapy.^14,15,23^ Prior to 2014, a number of clinical trials were conducted using autologous γδ T cells; however, only limited responses were reported.^12,26,27^ The main obstacles related to autologous γδ T-cell therapy include the difficulty of expansion, limited cell purity, and impaired cell functions. To obtain adequate cell numbers and optimal effector functions, we developed a NF consisting of ZOL (which selectively activates Vγ9Vδ2 γδ T cells), IL-2, IL-15, and vitamin C (Patent#: PCT/CN2019/075491) to better expand Vγ9Vδ2 T cells from healthy donors in vitro instead of using a gene modification-based methodology. Our results clearly indicated that the NF could promote γδ T-cell proliferation and differentiation, evidenced by significantly increased cell numbers and Ki-67^+^ proliferating cell numbers, a higher percentage of S-phase cells with a reduced percentage of G1-phase cells, and an elevated percentage of terminally differentiated effector memory (EMRA) cells. Moreover, new formula-expanded γδ T cells (NF cells) had a strikingly lower apoptosis rate than OF-expanded cells. In addition, NF cells expressed significantly higher levels of costimulatory molecules, which implied that the NF-expanded γδ T cells might promote antigen presentation to conventional CD4 and CD8 T cells, and thus correlate with superior antitumor activity. The substantially stronger cellular energy metabolism capability of NF cells was associated with their superior antitumor activity as well. Importantly, NF cells also expressed considerably higher levels of effector molecules (IFN-γ and TNF-α) and exhibited increased degranulation (induction of CD107a expression) associated with enhanced cytotoxicity against various cancer cell lines in vitro. Together, these results demonstrated that NF cells had optimal immune effector functions, as well as stronger in vitro antitumor activity than OF cells.

Furthermore, our preclinical in vivo experiments showed that adoptive transfer of NF cells significantly inhibited tumor growth in humanized mice transplanted with human lung tumor cells and prolonged tumor-bearing mouse survival from <3 months to as long as 8 months. It should be mentioned here that the humanized mouse model was established according to our published protocols^38,39^ and mice that survived GVHD caused by huPBMC pre-engraftment were used for experiments in our work. Notably, we observed that only NF cells visually colocalized with inoculated tumors, implying NF cells had a better ability to migrate to the tumor site than did OF cells. Although OF cells prolonged mouse survival as well, the inhibition of tumor growth was not as efficient as that induced by NF cells, as indicated by the significantly prolonged overall survival of NF-cell-treated mice. It should be remarked here that the variability in outcomes among the five mice per group could be partially related to the differences in immune reconstitution among individual mice or the differences in the antitumor activity of infused γδ T cells derived from different donors. Nevertheless, based on the present in vivo work, which was repeated once previously, we can conclude that NF cells have stronger antitumor activities than OF cells. In addition, although the Vγ9Vδ2 T cells used for adoptive transfer were not 100% pure (≥90%), no allogeneic effects were observed in our mouse experiments. Such results provided scientific support for the initiation of our clinical trials.

From our clinical trial investigations, we can draw some clinically important conclusions. First, allogeneic Vγ9Vδ2 T cells (NF cells) are clinically safe. This conclusion is strongly supported by our observation of 414 allogeneic NF-cell infusions in 132 cancer patients, which showed no significant adverse effects (e.g., immune rejection, cytokine storm, or GVHD effects). We emphasized here that the majority of patients received only 1–4 rounds of cell therapy and that the data obtained were thus only used to evaluate the clinical safety of allogeneic Vγ9Vδ2 T cells. It should be mentioned here that we measured cytokine release at 24 h post cell transfer therapy as well and found no evidence of cytokine storm after treatment with allogeneic Vγ9Vδ2 T cells. Nevertheless, a better time point for measuring cytokine release syndrome in patients with solid tumors would be ~1 week post cell therapy. Then, according to clinical observations, cell therapy could improve the quality of life in all patients, including pain relief, appetite, and sleep quality.

In our clinical study, among 132 cancer patients, only 8 liver and 10 lung cancer patients received ≥5 cell infusions; therefore, the obtained clinical data were used to preliminarily analyze the efficacy of allogeneic Vγ9Vδ2 T-cell therapy. NF-cell infusions (≥5) prolonged survival in seven of the eight liver cancer patients and eight of the ten lung cancer patients to ≥10 months. Quite excitingly, in our latest follow-up in June 2020, we observed that three liver and two lung cancer patients were still alive (corresponding to survival times between 30 and 35 months) with normal lives. This clearly demonstrates that allogeneic Vγ9Vδ2 T-cell therapy prolonged the survival of most late-stage cancer patients who received ≥5 cell infusions in this small cohort (18 patients) investigation, showing the clinical efficacy of this therapy. In addition, follow-up study of 18 patients preliminarily implied that liver cancer patients might have better clinical responses than lung cancer patients. However, such observations need to be verified by further increasing the patient numbers in extended clinical studies.

In conclusion, we developed a NF for the in vitro expansion of Vγ9Vδ2 T cells. The NF could significantly enhance cellular immune functions, including proliferation, differentiation, cellular energy metabolism, effector molecule expression, and cytotoxicity against cancer cell lines, while reducing the apoptotic rate in vitro. Moreover, an in vivo experiment using humanized mice firmly validated the superior antitumor cytotoxicity of NF cells compared with OF cells, which significantly prolonged mouse survival from <3 months to as long as 8 months. Most importantly, our clinical trials for the first time provided scientific evidence that allogeneic Vγ9Vδ2 T cells are clinically safe and preliminarily demonstrated therapeutic efficacy in solid tumor patients. Based on this work, we expect more clinical trials using allogeneic Vγ9Vδ2 T cells to treat malignant tumors to be conducted, which will eventually benefit tumor patients.

## Supplementary information

Figures 1–8

Supplementary Figure Legends

## Data Availability

Detailed clinical data sharing is not applicable to this article as clinical and commercial applications are ongoing.
