# Supplementary material for: Allogeneic Vγ9Vδ2 T-cell immunotherapy exhibits promising clinical safety and prolongs the survival of patients with late-stage lung or liver cancer
Source: Cell Mol Immunol. 2020 Sep 16;18(2):427–39. doi: 10.1038/s41423-020-0515-7 (PMC8027668; doi:10.1038/s41423-020-0515-7)
Supplement: Supplementary file 1 — Figures 1–8 [file 41423_2020_515_MOESM1_ESM.pptx]

## Slide 1
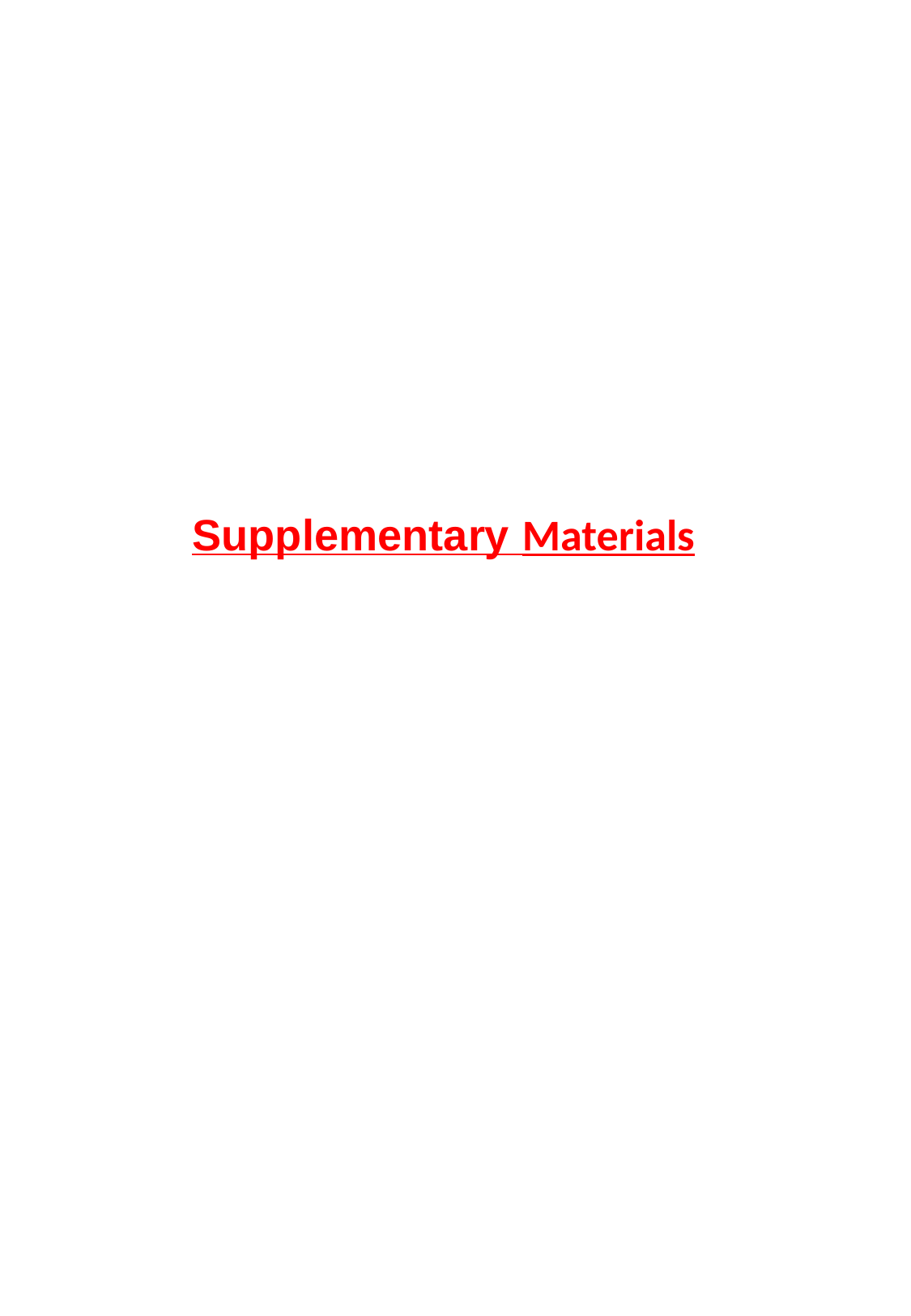

Supplementary Materials

## Slide 2
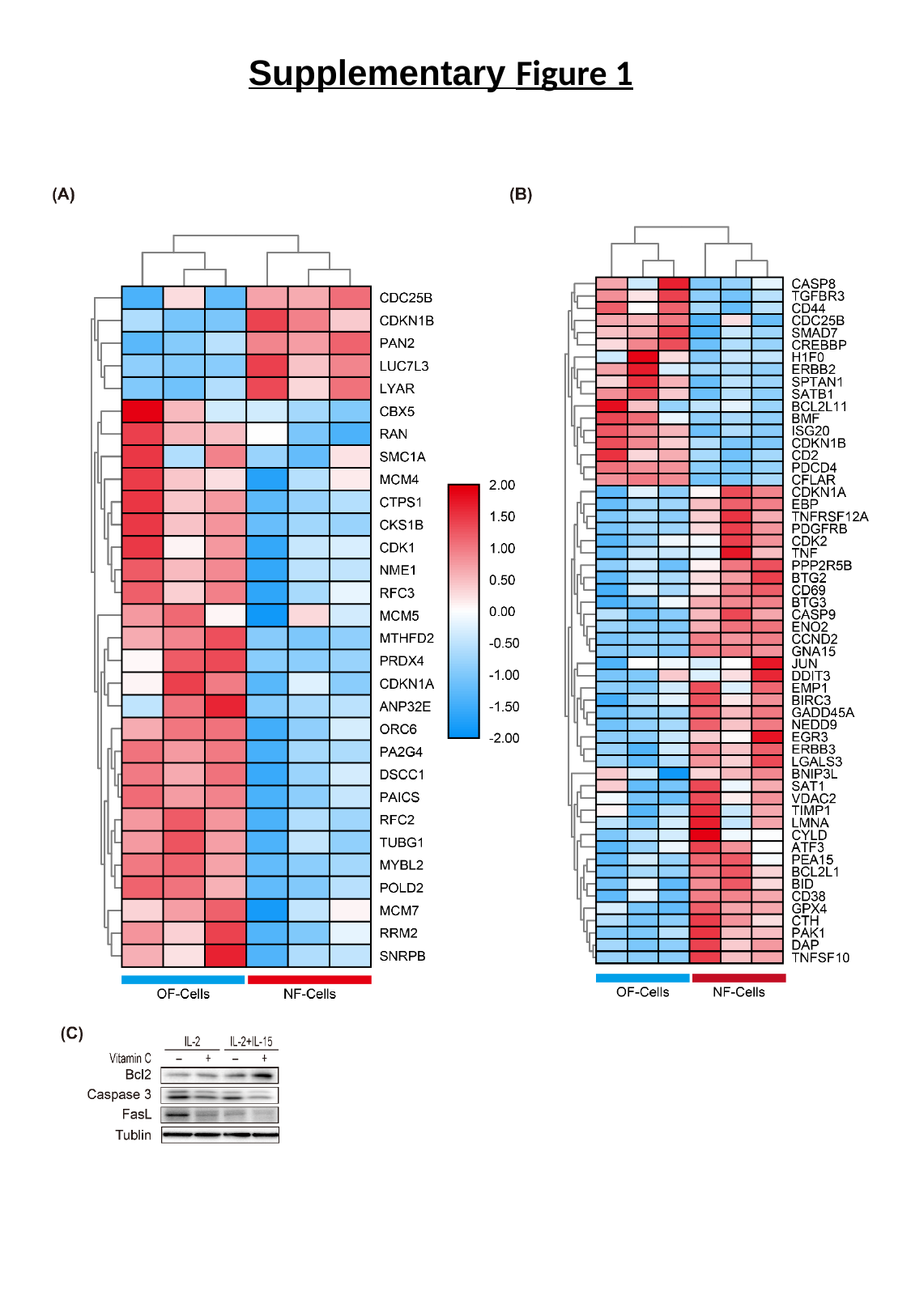

Supplementary Figure 1

## Slide 3
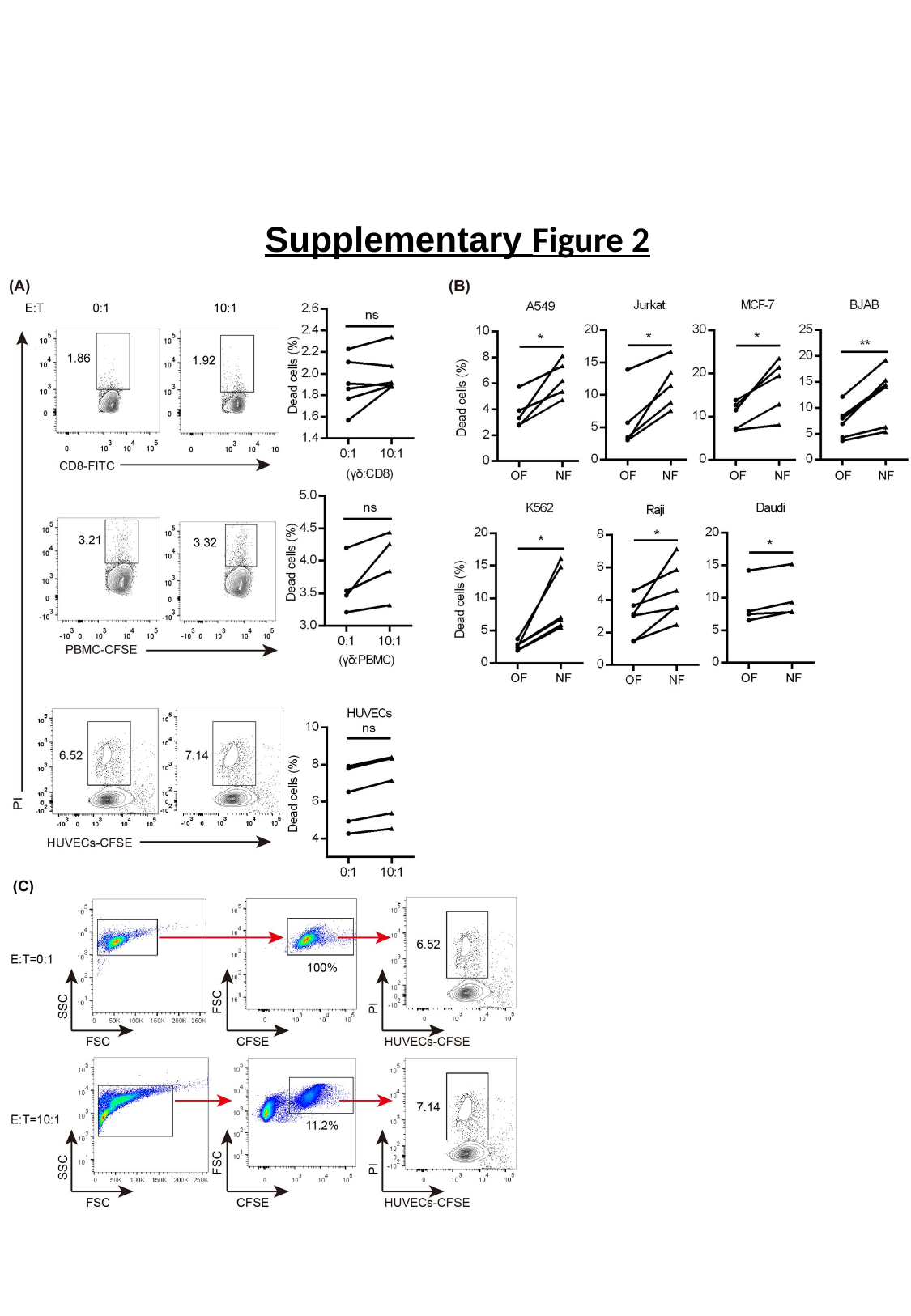

Supplementary Figure 2

## Slide 4
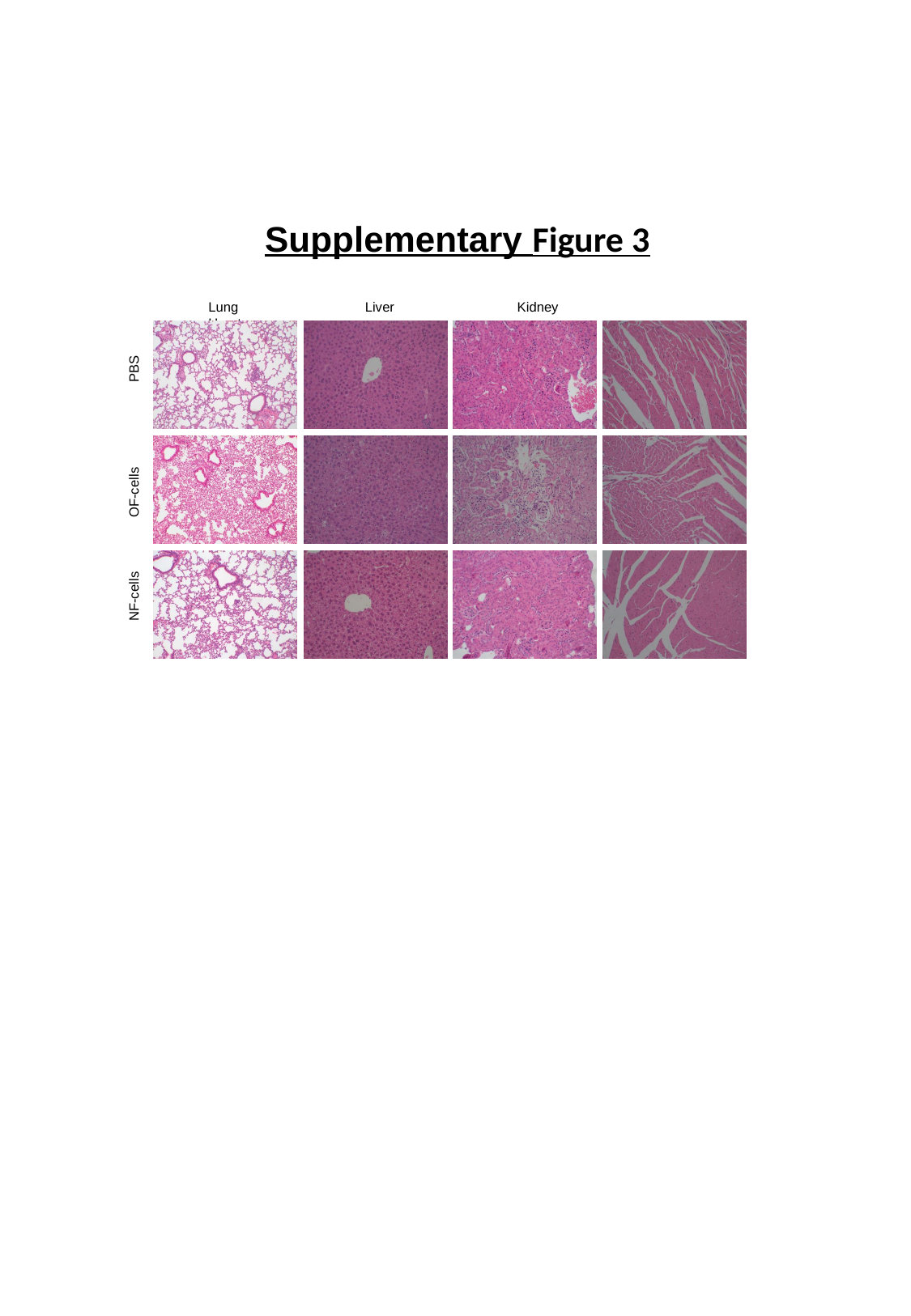

Supplementary Figure 3
Lung Liver Kidney Heart
NF-cells OF-cells PBS

## Slide 5
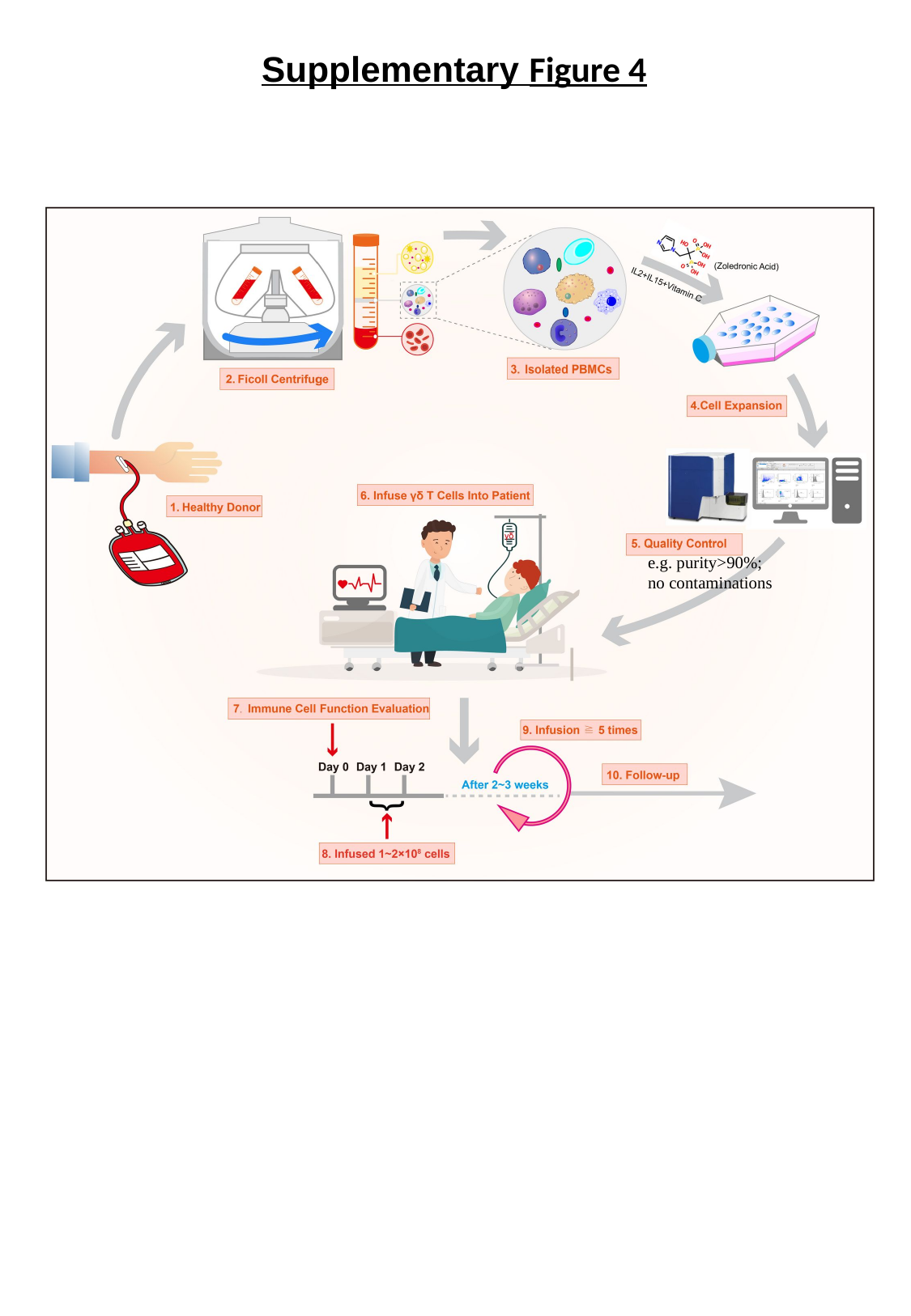

Supplementary Figure 4
e.g. purity>90%;
no contaminations

## Slide 6
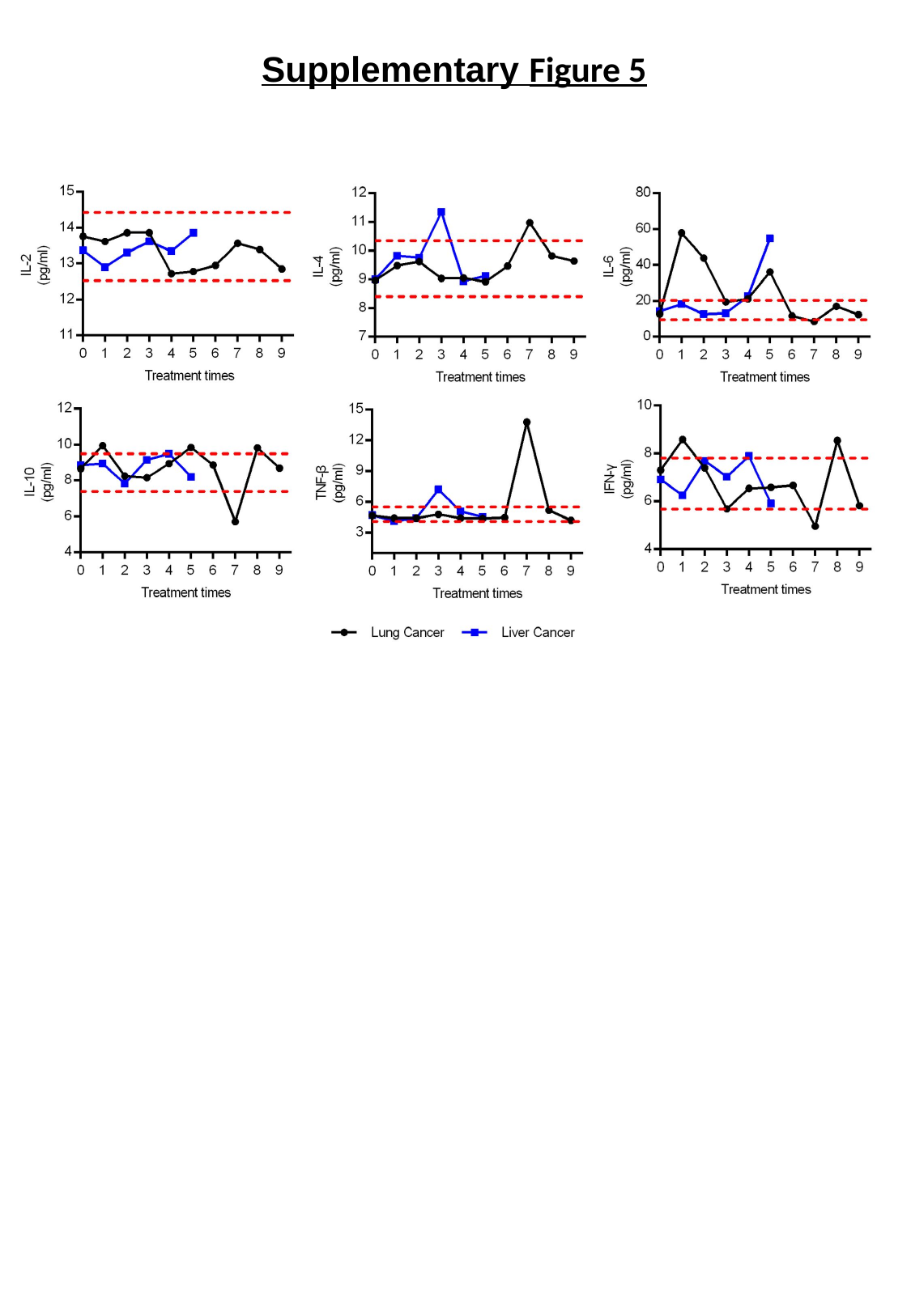

Supplementary Figure 5

## Slide 7
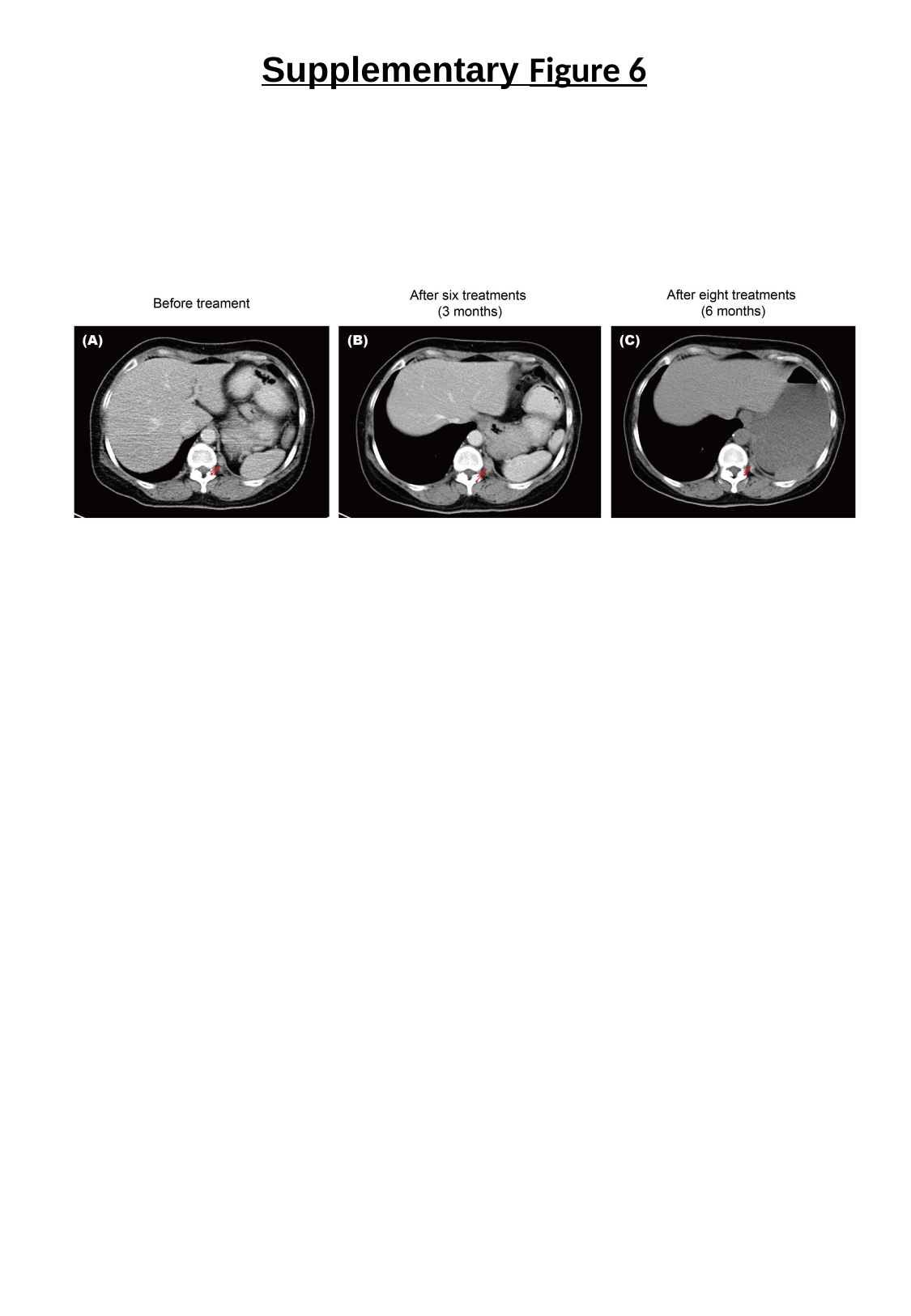

Supplementary Figure 6

## Slide 8
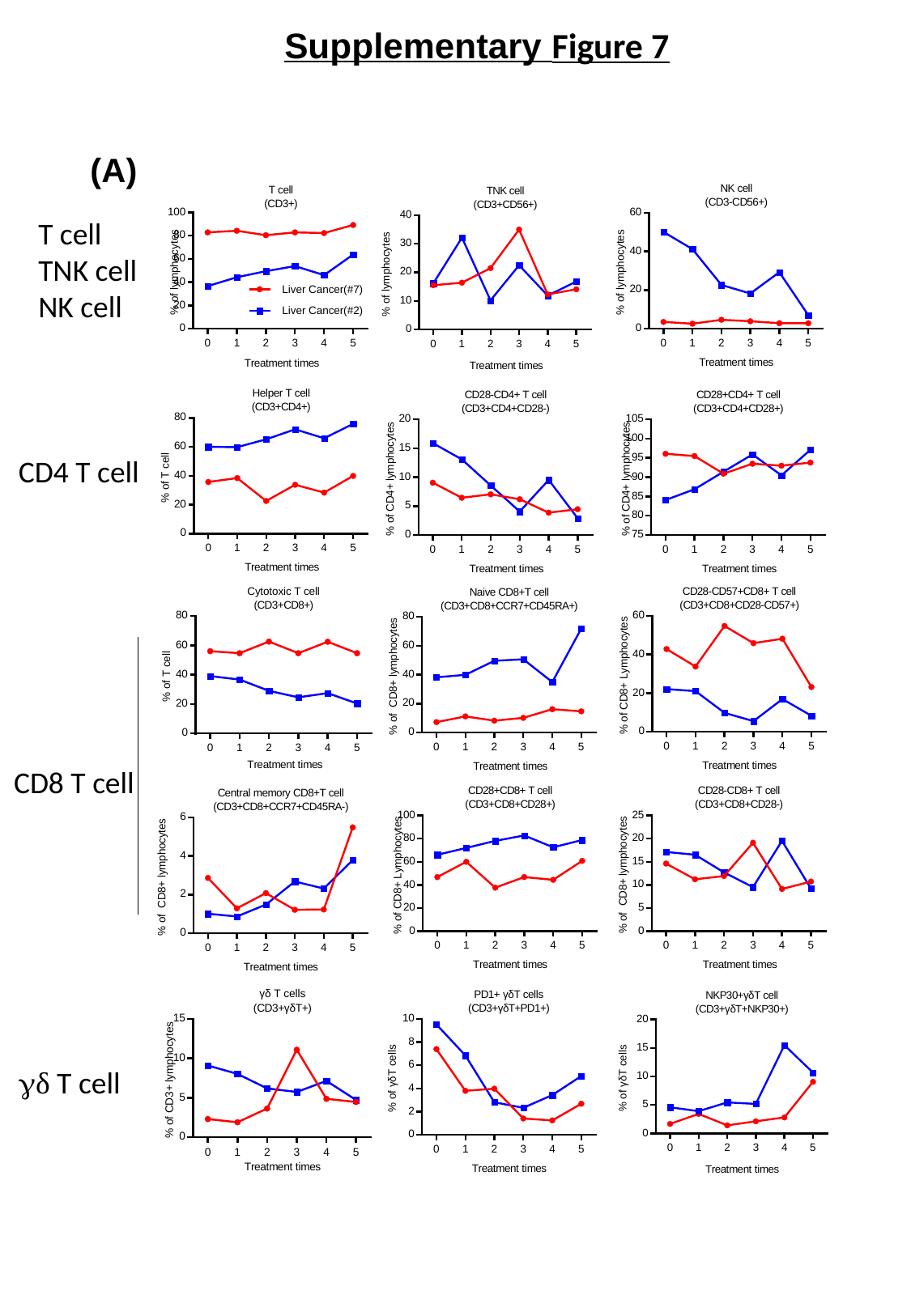

Supplementary Figure 7
(A)
T cell
TNK cell
NK cell
CD4 T cell
CD8 T cell
δ T cell

## Slide 9
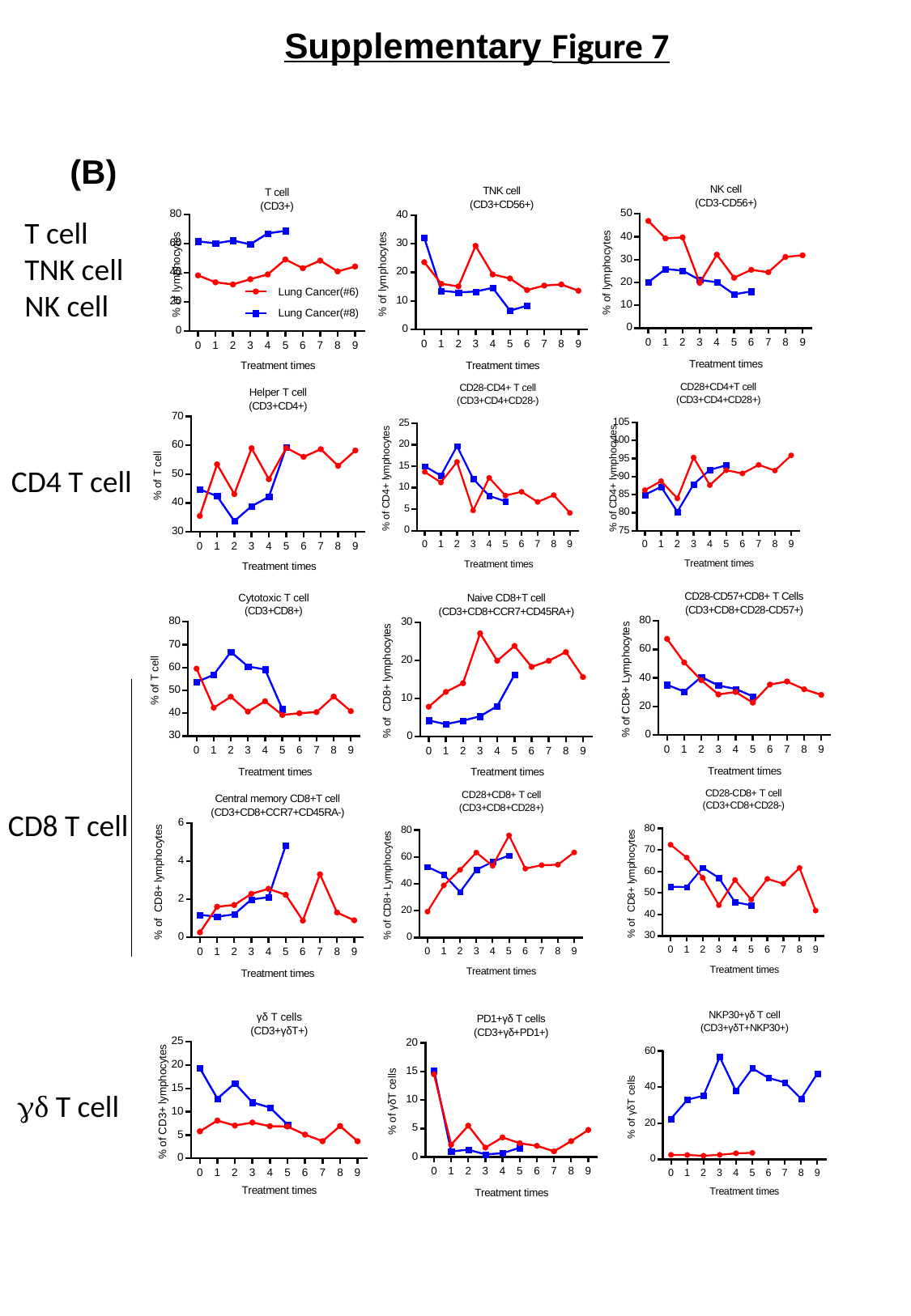

Supplementary Figure 7
(B)
T cell
TNK cell
NK cell
CD4 T cell
CD8 T cell
δ T cell

## Slide 10
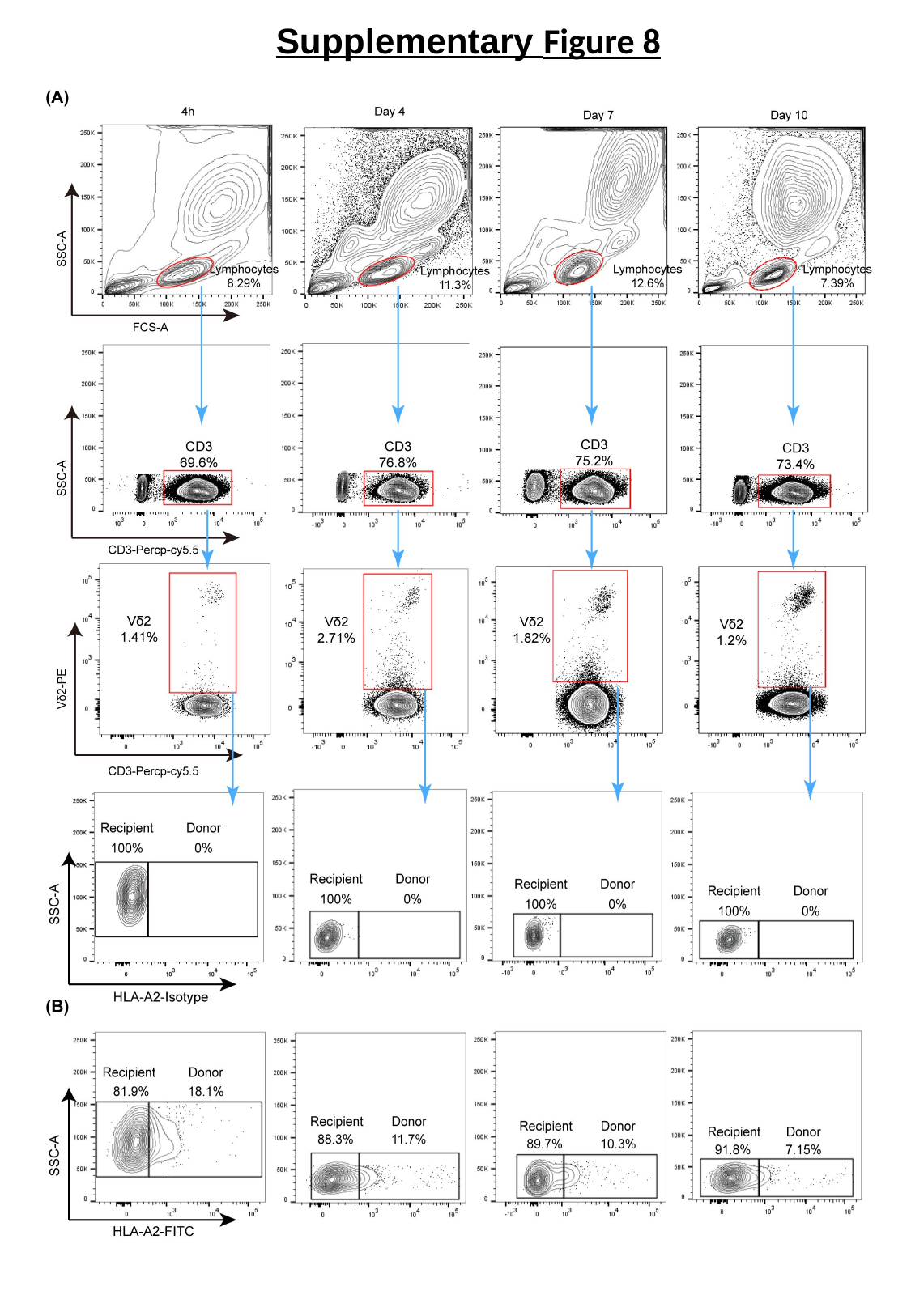

Supplementary Figure 8

## Slide 11
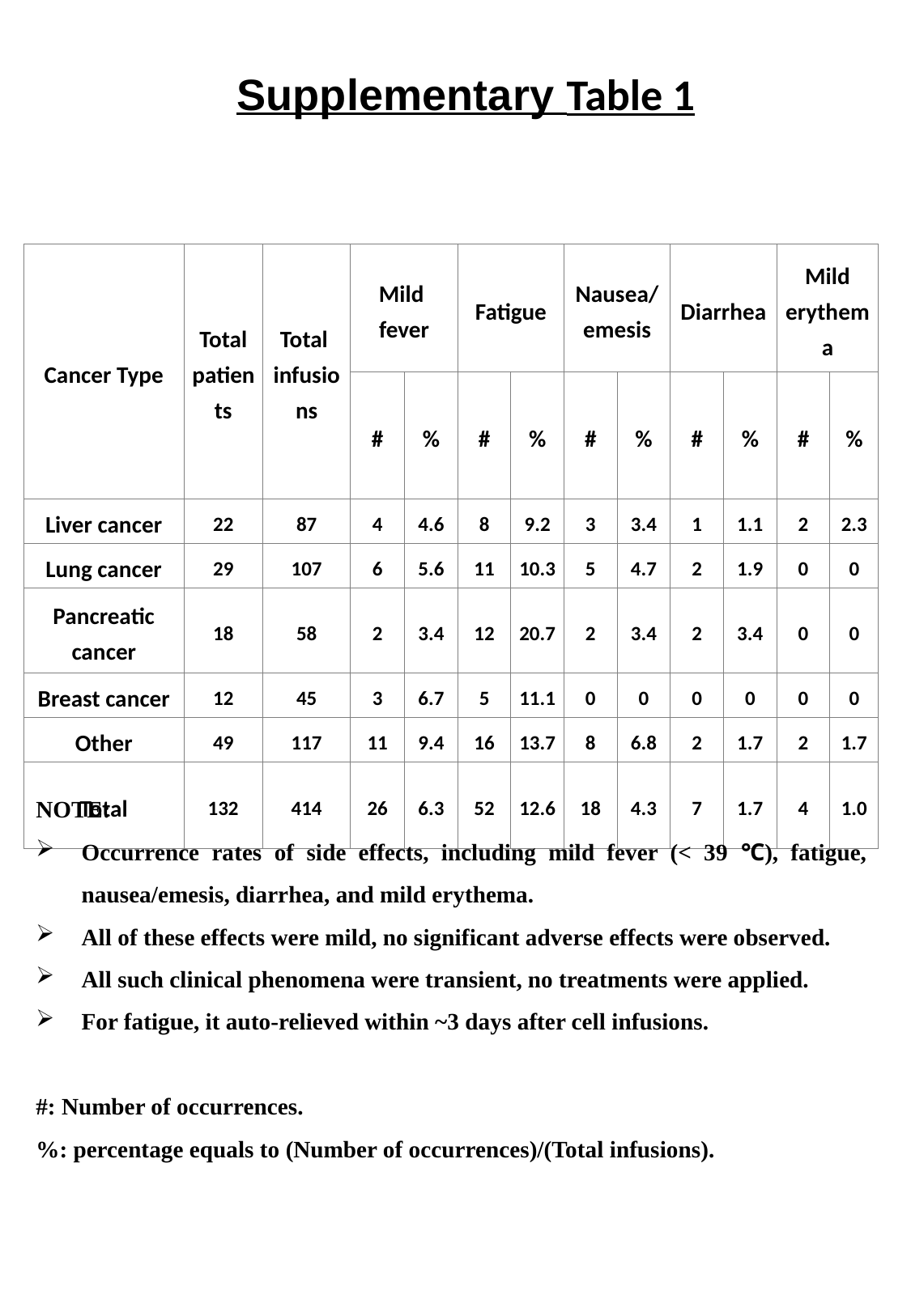

Supplementary Table 1
| Cancer Type | Total patients | Total infusions | Mild fever | | Fatigue | | Nausea/ emesis | | Diarrhea | | Mild erythema | |
| --- | --- | --- | --- | --- | --- | --- | --- | --- | --- | --- | --- | --- |
| | | | # | % | # | % | # | % | # | % | # | % |
| Liver cancer | 22 | 87 | 4 | 4.6 | 8 | 9.2 | 3 | 3.4 | 1 | 1.1 | 2 | 2.3 |
| Lung cancer | 29 | 107 | 6 | 5.6 | 11 | 10.3 | 5 | 4.7 | 2 | 1.9 | 0 | 0 |
| Pancreatic cancer | 18 | 58 | 2 | 3.4 | 12 | 20.7 | 2 | 3.4 | 2 | 3.4 | 0 | 0 |
| Breast cancer | 12 | 45 | 3 | 6.7 | 5 | 11.1 | 0 | 0 | 0 | 0 | 0 | 0 |
| Other | 49 | 117 | 11 | 9.4 | 16 | 13.7 | 8 | 6.8 | 2 | 1.7 | 2 | 1.7 |
| Total | 132 | 414 | 26 | 6.3 | 52 | 12.6 | 18 | 4.3 | 7 | 1.7 | 4 | 1.0 |
NOTE:
Occurrence rates of side effects, including mild fever (< 39 ℃), fatigue, nausea/emesis, diarrhea, and mild erythema.
All of these effects were mild, no significant adverse effects were observed.
All such clinical phenomena were transient, no treatments were applied.
For fatigue, it auto-relieved within ~3 days after cell infusions.
#: Number of occurrences.
%: percentage equals to (Number of occurrences)/(Total infusions).
