# Supplementary material for: Allogeneic Vγ9Vδ2 T-cell immunotherapy exhibits promising clinical safety and prolongs the survival of patients with late-stage lung or liver cancer
Source: Cell Mol Immunol. 2020 Sep 16;18(2):427–39. doi: 10.1038/s41423-020-0515-7 (PMC8027668; doi:10.1038/s41423-020-0515-7)
Supplement: Supplementary file 2 — Supplementary Figure Legends [file 41423_2020_515_MOESM2_ESM.docx]

**Supplementary Figure Legends**

**Supplementary Figure 1.**

**Effects of the new formula (NF) on the proliferation, differentiation and apoptosis of Vγ9Vδ2 T cells.** **(A-B)** Heatmaps of differentially expressed genes associated with proliferation **(A)** or apoptosis **(B)**. The heatmaps were generated from the log 2 expression of counts normalized to transcript size and million mapped read (FPMK) values. Differentially expressed genes (DEGs) are listed along the Y axis in the order they clustered in, as indicated by the colored line along the Y axis. Each column contains expression values for an individual bird, with groups indicated along the X axis, and clustering indicated by the dendrogram above the figure. Deeper red colors indicate higher upregulation of gene expression, while deeper blue colors indicate lower downregulation. **(C)** Western blot results (original WB scan image). The expression of the antiapoptotic gene Bcl2 was significantly upregulated in IL-2+IL15+vitamin C cells (NF-cells), while the levels of the apoptosis genes Fas-L and Caspase 3 were noticeably reduced in NF-cells compared with IL-2-treated cells (OF-cells).

**Supplementary Figure 2.**

**Cytotoxicity of Vγ9Vδ2 T cells against normal cells and tumor cells. (A)** Vγ9Vδ2 T cells did not kill CD8^+^ T cells or PBMCs. CD8^+^ T cells were isolated from PBMCs with anti-human CD8 magnetic particles (BD). PBMCs and HUVECs were labeled with CFSE and then cocultured with allogeneic NF-Vγ9Vδ2 T cells for 24 h at different E:T (γδ:CD8; γδ:PBMC) ratios (0:1 (control group) and 10:1). The CD8^+^ T cells, PBMCs and NF-cells were from unrelated donors. An anti-CD8 fluorophore-conjugated antibody and propidium iodide (PI) were used to identify dead CD8^+^ T cells. The death of CD8^+^ T cells and dead PBMCs are shown as the percentage of PI^+^ cells in the CD8^+^ population or CFSE^+^ population. **(B)** Results for γδ T cell killing of cancer cells at a low E:T ratio (1:1). The death of CFSE-labeled A549, Jurkat, MCF-7, BJAB, K562, Raji and Daudi target cells was analyzed after 6 hr of coculture with Vγ9Vδ2-T cells by PI staining and flow cytometry. Each linked line between OF and NF in all graphs represents γδ T cells from an individual donor. **(C)** Representative example graphs showing the flow cytometry gating strategy.

**Supplementary Figure 3.**

**Representative histological images of hematoxylin and eosin-stained lungs, livers, kidneys, and hearts from tumor-bearing humanized mice.** NF-cell treatment had no side effects on these organs (magnification: 10×).

**Supplementary Figure 4.**

**Schematic diagram for allogeneic Vγ9Vδ2 T cell expansion and infusion.** One hundred milliliters of peripheral blood was collected from healthy donors (step 1). PBMCs were isolated (steps 2 and 3), and γδ T cells were cultured (step 4) for approximately 14 days *in vitro*. After a quality check by immunofluorescence labeling plus flow cytometry analysis and pathogen testing (step 5), γδ T cells were intravenously infused (1~2×10^8^ cells per treatment) into patients (step 6). Patients received only one γδ T cell infusion on day 1 or day 2 (step 8), followed by infusion every 2~ 3 weeks for the first five treatments (step 9) and then one treatment every 30-60 days thereafter. Patient immune cell function was also analyzed (step 7) before and after γδ T cell treatments by analyzing peripheral immune phenotypes using flow cytometry. Only those patients who received more than 5 infusions were followed and further analyzed (step 10).

**Supplementary Figure 5.**

**Clinical safety evaluation through cytokine examination in a lung cancer patient (patient #6 in Table 1) and a liver cancer patient (patient #2 in Table 1) after cell therapy.** According to clinical observations, allogeneic Vγ9Vδ2 T cells did not induce significant cytokine storm. Serum was collected at 24 hours post γδ T cell infusion, and the serum levels of inflammatory cytokines were measured using the BD CBA Human Inflammation Kit at different time points. Cytokines were analyzed at different time points, as shown in figures (number ‘0, 1, 2, 3, 4, 5, 6, 7, 8, 9’). The dashed red line indicates the normal range, and the parameters for the normal range have been used in clinical practice for years. Black curve: lung cancer patient; blue curve: liver cancer patient.

**Supplementary Figure 6.**

**Computed tomography (CT) scans of a lung cancer patient (patient #6 in Table 1) performed at 3 time points after eight treatments with allogeneic γδ T cells.** Before receiving allogeneic γδ T cell therapy, the patient developed intraperitoneal metastasis of lung cancer. The red arrow shows a swollen lymph node in the left posterior of the lower mediastinum **(A)**. No newly visible lesion in the left posterior region was observed compared with a previous abdominal CT scan after six **(B)** and eight **(C)** treatments with allogeneic γδ T cells.

**Supplementary Figure 7.**

**Immune cell parameters were assessed in liver cancer (patients #2 and #7 in Table 1) (A) and lung cancer (patients #6 and #8 in Table 1) (B) patients before and after cell therapy.** The immune phenotypes of the patients were analyzed before and after γδ T cell treatment**.** Five milliliters of peripheral blood was extracted from each patient each time, including 1-3 days before receiving Vγ9Vδ2 T cell treatment. Peripheral blood mononuclear cells (PBMCs) were isolated by Ficoll density gradient centrifugation and stained with the indicated antibodies. The results for one lung cancer patient and one liver cancer patient are shown. Then, fluorophore-conjugated antibody-labeled cells were analyzed using flow cytometry.

**Supplementary Figure 8.**

**HLA-A2-based tracking of** **allogeneic γδ T cells in a patient before and after NF-cell infusion.** Allogeneic γδ T cells from healthy donors were tracked to identify their persistence *in vivo* by discriminating HLA-A2 molecules (BD Biosciences, clone: BB7.2). NF-cells from one HLA-A2-positive donor were adoptively transferred into one HLA-A2-negative recipient. PBMCs were isolated from the patient periodically and analyzed by flow cytometry for HLA-A2-positive cells. In this representative example, the percentage of allogeneic γδ T cells in the patient gradually decreased from 18.7% at 4 hours to 7.23% on day 10. **(A)** Flow cytometry gating strategy based on an isotype control. **(B)** Representative flow graphs for the HLA-A2 analysis.

**Supplementary Table 3**.

**Summary of clinical safety observations for allogeneic Vγ9Vδ2 T cell infusions.** No significant adverse effects (e.g., immune rejection, cytokine storm, or GVHD effects) were observed in 132 patients with a total number of 414 infusions.
